# Sensitivity Enhancement of Transition Metal Dichalcogenides/Silicon Nanostructure-based Surface Plasmon Resonance Biosensor

**DOI:** 10.1038/srep28190

**Published:** 2016-06-16

**Authors:** Qingling Ouyang, Shuwen Zeng, Li Jiang, Liying Hong, Gaixia Xu, Xuan-Quyen Dinh, Jun Qian, Sailing He, Junle Qu, Philippe Coquet, Ken-Tye Yong

**Affiliations:** 1 School of Electrical and Electronic Engineering, Nanyang Technological University, Singapore, 639798; 2 CINTRA CNRS/NTU/THALES, UMI 3288, Research Techno Plaza, 50 Nanyang Drive, Border X Block, Singapore, 637553; 3 State Key Laboratory of Modern Optical Instrumentation, Centre for Optical and Electromagnetics Research, Zhejiang University, Hangzhou 310058, China; 4 Key Laboratory of Optoelectronics Devices and Systems of Ministry of Education/Guangdong Province, College of Optoelectronic Engineering, Shenzhen University, Shenzhen, P. R. China; 5 Institut d’Electronique, de Microélectronique et de Nanotechnologie (IEMN), CNRS UMR 8520 – Université de Lille 1, 59650 Villeneuve d’Ascq, France

## Abstract

In this work, we designed a sensitivity-enhanced surface plasmon resonance biosensor structure based on silicon nanosheet and two-dimensional transition metal dichalcogenides. This configuration contains six components: SF10 triangular prism, gold thin film, silicon nanosheet, two-dimensional MoS_2_/MoSe_2_/WS_2_/WSe_2_ (defined as MX_2_) layers, biomolecular analyte layer and sensing medium. The minimum reflectivity, sensitivity as well as the Full Width at Half Maximum of SPR curve are systematically examined by using Fresnel equations and the transfer matrix method in the visible and near infrared wavelength range (600 nm to 1024 nm). The variation of the minimum reflectivity and the change in resonance angle as the function of the number of MX_2_ layers are presented respectively. The results show that silicon nanosheet and MX_2_ layers can be served as effective light absorption medium. Under resonance conditions, the electrons in these additional dielectric layers can be transferred to the surface of gold thin film. All silicon-MX_2_ enhanced sensing models show much better performance than that of the conventional sensing scheme where pure Au thin film is used, the highest sensitivity can be achieved by employing 600 nm excitation light wavelength with 35 nm gold thin film and 7 nm thickness silicon nanosheet coated with monolayer WS_2_.

Surface plasmon resonance (SPR)-based biosensors have attracted great attention as one of the leading optical sensing technologies during the last two decades due to their unique advantages such as real-time and label-free detection^1^,^2^,^3^. They played an important role in monitoring various biomolecular interactions like protein bindings and DNA hybridization^4^,^5^. Further applications such as pharmacology and early disease diagnostics are promising if the SPR sensitivities have a drastic improvement^6^. The first observation of the SPR dated back to 1902, however, the complete explanations on this phenomenon were not provided by the scientific community until 1968 when Kretschmann, Raether and Otto proposed effective excitation configurations and abundant theoretical demonstration^7^,^8^. The surface plasmons (SPs) can be considered as electron density waves that propagate at the interface between metal and dielectric. In order to excite the surface plasmon waves (SPW), the incident photons or electrons should oscillate with the free electrons on the metal surface to form the resonances. Only *p*-polarized light (TM waves) contributes to the excitation of the SPs, while *s*-polarized light (TE waves) could act as the reference signals. When the horizontal component of the incident wave vector *k*_*x*_ (i.e., the evanescent wave vector) matches with the surface plasmon wave vector *k*_*sp*_, the surface plasmon resonance phenomenon occurs, as shown in equation (1),




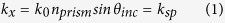




where *k*_*0*_ represents the incident wave vector in free space and *θ*_*inc*_ denotes the incident angle. Here, the incident angle is known as the resonance angle (or SPR angle). When the excitation light wavelength was fixed, one can obtain a SPR curve with a dip by scanning the incident angle and monitoring the reflectivity. The minimum of the reflectivity (nearly zero) corresponded to the reflected intensity at the resonance angle. When the SPW was excited, part of the incident optical energy was converted into the SPW resonance energy, leading to the drastic decrease in the reflected intensity. Ideally, all incident energy should be absorbed to support the resonant oscillations and result in a strong evanescent field. For the SPR biosensor, the SPR angle serves as an important output signal for the angular interrogation system. Since the refractive index changes of the sensing layer that are induced by the adsorption of biomolecules on the sensing surface would lead to a redistribution of SPR electromagnetic field, a significant SPR angle shift could be obtained and collected through an optical detector. This unique characteristic enables SPR biosensors to show excellent performances for real-time and label-free detections. However, the sensitivity is known to be limited especially when the weight of the biomolecules analyte is less than 500 Da^9^. Various methods are provided to enhance the sensitivity: Silver thin film as SPR sensing substrate was demonstrated to have better performance than that with gold in sensitivity, however the weak chemical stability of silver impedes its further development^6^; In addition, the coating of an additional dielectric nanolayer on the sensing film was reported to exhibit sensitivity enhancement effects^5^. Attributed to the excellent optoelectronic properties and the advanced fabrication techniques (e.g. graphene growth on metallic substrates)^10^,^11^, graphene layers have been employed to enhance the SPR biosensor sensitivity. As reported in Wu *et al.*^12^ study, 10 layers of the graphene coated on the gold sensing surface can improve the sensitivity by 25%. The enhanced effect of single nanomaterial toppings, however, still seems insufficient for further development of SPR biosensor applications. Thus, hybrid nanostructures such as silicon-graphene and MoS_2_-graphene thin film were investigated and revealed prominent sensitivity enhanced effect^13^,^14^,^15^. The silicon nanosheet is able to enhance the sensitivity of the SPR biosensor because of its large real value of the refractive index. It also serves as a protective layer of metal film to improve the overall system stability^16^,^17^. Recently, the emerging two-dimensional (2D) transition metal dichalcogenides (TMDCs) have been widely used in transistors and photodetectors due to the remarkable electrical and optical properties. The TMDCs family consists of more than forty compounds that generally defined as MX_2_, where M stands for the transition metal from group IV to group VII, like Nb, Ta, Mo and W; and the X denotes the chalcogen such as S, Se and Te. Monolayer MX_2_ contains three atomic layers where the transition metal layer is sandwiched by two chalcogens layers. Each layer is stacked via van der Waals forces. In this work, we focus on the group-IV semiconductor dichalcogenides MoX_2_ and WX_2_, namely Molybdenum disulfide (MoS_2_), Molybdenum diselenide (MoSe_2_), Tungsten disulfide (WS_2_) and Tungsten diselenide (WSe_2_). The rapid fabrication development of the high quality (i.e., large areas, highly uniform) individual 2D MX_2_ layers by chemical exfoliation method promotes versatility of 2D MX_2_ in various fields, such as photonics, electronics, energy storage, catalysis and even biomedical applications^18^,^19^,^20^. Although the properties of bulk MX_2_ have been investigated for decades, the successful translation of 2D MX_2_ in optoelectronics and nanoelectronics is still remained in a stagnant stage. It is well known that when the bulk material is downscaled to a single layer, the bandgap transition would be gradually shifted from indirect to direct state^20^,^21^. This can be explained by the quantum confinement and resulted from the change in hybridization between orbital of Mo/W and X atoms^20^,^22^. The electronic bands of these monolayer materials are comparable to that of silicon (1.1 eV), which allows good performance in digital transistors^23^. Furthermore, these characteristics also affect the photophysical properties. For the semiconductor materials with a direct bandgap like MoX_2_ and WX_2_, the photons can be directly absorbed or emitted if the external energy is larger than the bandgap. However, for the indirect bandgap materials, photons could not be absorbed directly, since additional phonons were needed to provide the energy for the electron to surpass the intermediate state and transfer the momentum to the crystal lattice. Therefore, the photon absorption process for direct bandgap materials are much more efficient than that of indirect bandgap materials.

The single layered MoS_2_, which known as “beyond graphene” 2D nanocrystals material has attracted a great deal of attention. Due to the quantum confinement effects, the monolayer MoS_2_ has a direct bandgap of 1.8 eV, while bulk MoS_2_ has an indirect bandgap of 1.2 eV^22^,^24^. This allows 2D MoS_2_ to be used in nano-transistor channel with a large switching ratio^25^ and in photodetectors with a high responsivity up to 5 × 10^8^ AW^−1 ^26. Monolayer MoS_2_ also plays a key role for enhancing the sensitivity of SPR optical sensor^13^. Basically, a MoS_2_ enhanced hybrid nanostructure SPR biosensor can drastically improve detection limit of the device by using phase modulation technique. Similar to MoS_2_, the confinement of charge carriers on the horizontal atomic plane can gradually enlarge the energy gap of the WS_2_ atomic layers^27^,^28^,^29^. Theoretical studies show that monolayer WS_2_ has better performance compared to MoS_2_ in terms of enhancing the carrier mobility when they serve as channels in the transistor. This is attributed to the lower electron effective mass of WS_2_ compared to other MX_2_ materials^30^. In the research field of photoelectronics, nano-scaled WS_2_ also showed outstanding performance. For example, 2D heterostructures consisting of WS_2_, MoS_2_, GaSe and graphene can exhibit photovoltaic effects with external quantum efficiencies up to 30%^27^,^31^. Moreover, the employment of WS_2_ monolayer in plasmonic applications has enhanced the efficiency appreciably^32^. WSe_2_ nanolayers also attracted wide attention in photoelectronics, due to its fine absorption and emission features^33^ as well as the strong exciton charging effect^34^. Koperski *et al.*^35^ reported a comprehensive study of optical micro-spectroscopy based on thin layers of WSe_2_, where narrow emission lines (~100 μeV linewidth) were obtained because the monolayer WSe_2_ can generate luminescence within the same energy range. Ross *et al.*^36^ demonstrated a monolayer WSe_2_ p–n junction based LED structure that produced effective injections of electrons and holes due to the high optical quality. It yielded bright electroluminescence with much smaller injection current and linewidth compared with MoS_2_-based structures. The monolayer MoSe_2_ also provides promising optical applications because of its direct bandgap (identified at 1.55 eV)^37^. It is reported that the MoSe_2_ nanostructures show reversible and sensitive photo-responsive (PR) properties with the PR current values reaching up to 2.55 × 10^−5^ A^38^. The lateral heterojunctions within monolayer MoSe_2_-WSe_2_ are visible under the optical microscope and show enhanced photoluminescence^39^. The strong photoluminescence emission is caused by the transition from an indirect band gap semiconductor of bulk material to a direct band gap semiconductor in atomically thin form^40^. These properties provided a solid foundation for atomic thin semiconductor dichalcogenides as promising candidates for next generation nanoelectronics, and optoelectronics^41^.

Based on these remarkable properties of silicon and group-IV semiconductor dichalcogenides, we propose a new configuration for sensitivity enhanced SPR biosensors based on silicon-MX_2_ heterostructures. As shown in Fig. 1, based on the Kretschmann attenuated total reflection (ATR) configurations, gold thin film is attached at the bottom of the SF10 prism followed by silicon nanosheet and 2D MX_2_. The 2D MX_2_ layers which are directly contacted with biomolecular analyte have dual effects: (i) as the signal-enhanced layer due to a high charge transfer efficiency from the MX_2_ surface to the Au surface^42^,^43^,^44^; (ii) as sensing platform to capture the biomolecules through the van der Waals interaction^45^,^46^.

## Results and Discussion

In the ATR configuration, according to the principle of energy conversation the sum of the absorption *A*, reflectance *R* and transmittance *T* must be equal to 1 (i.e., *A* + *R* + *T* = *1*) assuming no energy loss besides the materials absorption. Under the ATR condition, *T* is always equal to zero, hence the absorption of the system can be reduced to *A* = 1 − *R*. When the SPs were excited, the reflectance *R* gradually decreased until it reached a minimum. The minimum *R* was close to zero indicating that the incident energy was almost completely absorbed by the layered materials. Therefore, maximum incident light energy transfer to the evanescent wave is required in order to achieve the best SPR enhancement performance^4^,^15^,^47^,^48^.

### Optimization of number of MX_2_ layers

**Figure 2:**
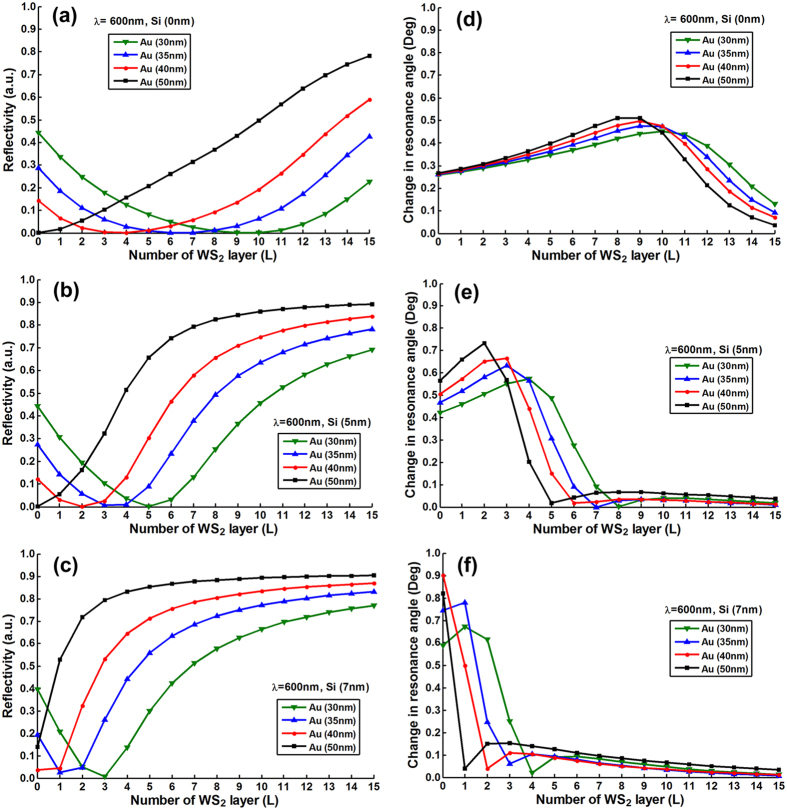
The minimum reflectivity in SPR curve as a function of the number of WS_2_ layers at 600 nm excitation wavelength with various thickness of the gold thin film and silicon nanosheet (**a**) 0 nm (**b**) 5 nm (**c**) 7 nm; and the corresponding changes in the resonance angle for a fixed refractive index change in the biomolecular analyte (*∆n*_*bio*_ = 0.005) as a function of the number of layers of WS_2_ at 600 nm excitation wavelength with various thickness of gold thin film and silicon nanosheet (**d**) 0 nm (**e**) 5 nm (**f**) 7 nm.

**Figure 3:**
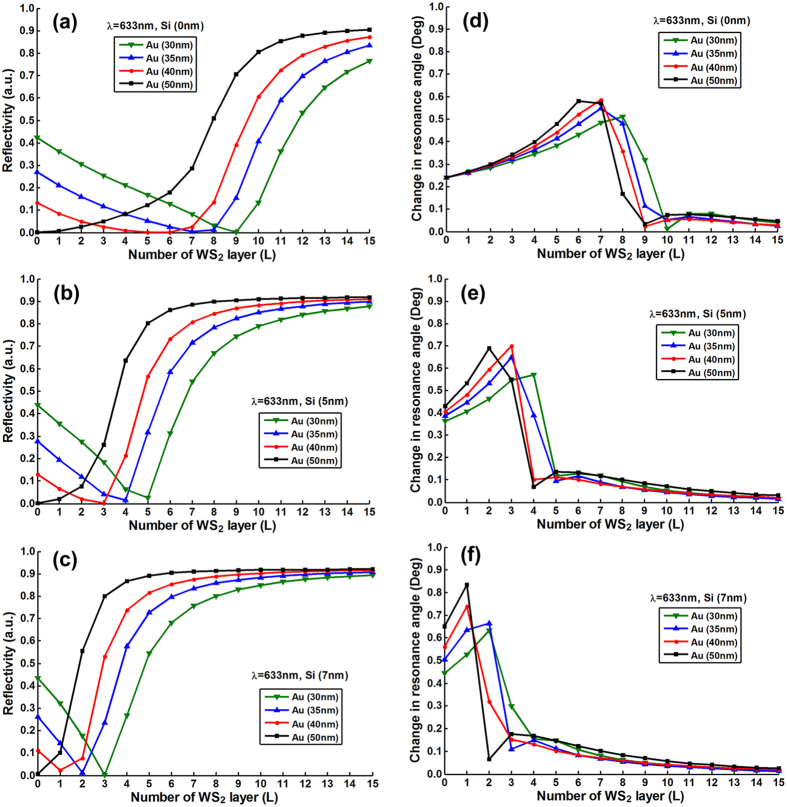
The minimum reflectivity in SPR curve as a function of the number of WS_2_ layers at 633 nm excitation wavelength with various thickness of the gold thin film and silicon nanosheet (**a**) 0 nm (**b**) 5 nm (**c**) 7 nm; and the corresponding changes in the resonance angle for a fixed refractive index change in the biomolecular analyte (*∆n*_*bio*_ = 0.005) as a function of the number of layers of WS_2_ at 633 nm excitation wavelength with various thickness of gold thin film and silicon nanosheet (**d**) 0 nm (**e**) 5 nm (**f**) 7 nm.

**Figure 4:**
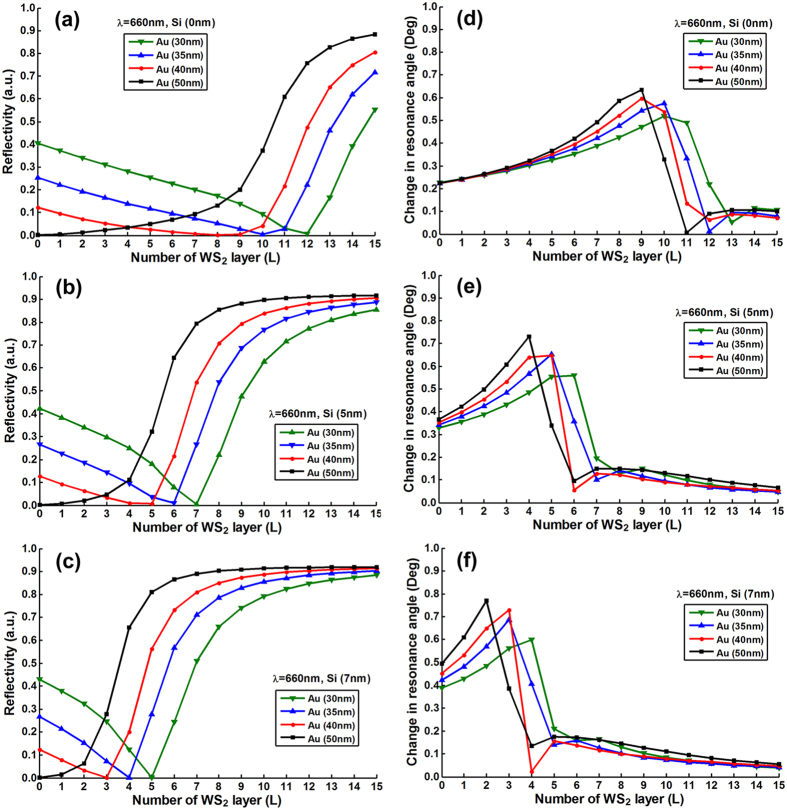
The minimum reflectivity in SPR curve as a function of the number of WS_2_ layers at 660 nm excitation wavelength with various thickness of the gold thin film and silicon nanosheet (**a**) 0 nm (**b**) 5 nm (**c**) 7 nm; and the corresponding changes in the resonance angle for a fixed refractive index change in the biomolecular analyte (*∆n*_*bio*_ = 0.005) as a function of the number of layers of WS_2_ at 660 nm excitation wavelength with various thickness of gold thin film and silicon nanosheet (**d**) 0 nm (**e**) 5 nm (**f**) 7 nm.

**Figure 5:**
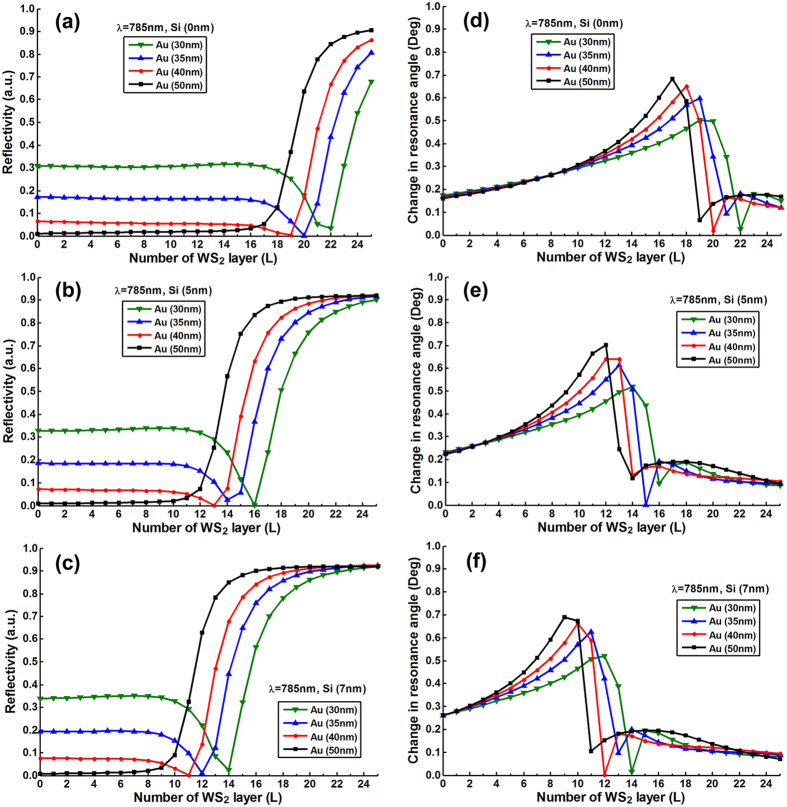
The minimum reflectivity in SPR curve as a function of the number of WS_2_ layers at 785 nm excitation wavelength with various thickness of the gold thin film and silicon nanosheet (**a**) 0 nm (**b**) 5 nm (**c**) 7 nm; and the corresponding changes in the resonance angle for a fixed refractive index change in the biomolecular analyte (*∆n*_*bio*_ = 0.005) as a function of the number of layers of WS_2_ at 785 nm excitation wavelength with various thickness of gold thin film and silicon nanosheet (**d**) 0 nm (**e**) 5 nm (**f**) 7 nm.

**Figure 6:**
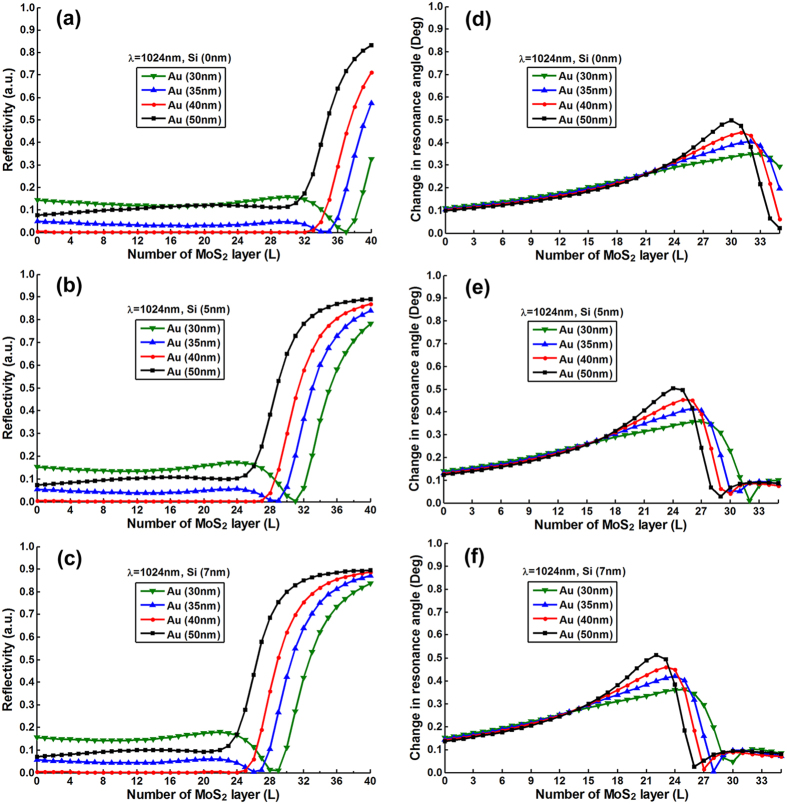
The minimum reflectivity in SPR curve as a function of the number of WS_2_ layers at 1024 nm excitation wavelength with various thickness of the gold thin film and silicon nanosheet (**a**) 0 nm (**b**) 5 nm (**c**) 7 nm; and the corresponding changes in the resonance angle for a fixed refractive index change in the biomolecular analyte (*∆n*_*bio*_ = 0.005) as a function of the number of layers of WS_2_ at 1024 nm excitation wavelength with various thickness of gold thin film and silicon nanosheet (**d**) 0 nm (**e**) 5 nm (**f**) 7 nm.

To optimize the number of MX_2_ layers, we plotted the resonance depths (i.e., the value of minimum reflectivity) change as a function of number of MX_2_ layers with various thickness of silicon nanosheet (i.e., 0 nm, 5 nm, 7 nm) and gold thin film (i.e., 30 nm, 35 nm, 40 nm, 50 nm). Figures 2a–c, 3a–c, 4a–c, 5a–c and 6a–c show the simulation results where different excitation wavelengths at 600 nm, 633 nm, 660 nm, 785 nm and 1024 nm were used. In general, two features for the SPR curves were observed: (i) When the silicon thickness was fixed and decreasing the gold thickness, the SPR dips redshifted with larger number of MX_2_ layers; similarly, when the thickness of gold thin film was fixed and decreasing the thickness of silicon, the reflectivity dips would also redshifted with the larger number of MX_2_ layers. This indicates that both silicon and MX_2_ layers can serve as absorption mediums and transfer energy to the gold thin film. (ii) It is worth noting that when the gold thickness was fixed at 50 nm, no MX_2_ is required to achieve the minimum reflectivity dip since 50 nm is the optimized thickness of gold thin film based on the conventional Kretschmann ATR configuration^6^. By analyzing the relationship between reflectivity and the number of MX_2_ layers, optimization of the number of MX_2_ layers was possible by selecting the specific number of layers that corresponded to the minimum resonance depths (i.e., the value of reflectivity that closest to zero). The detailed optimized number of MX_2_ layers with minimum reflectivity *minR* less than 0.03 were listed in Tables S1–S20 (Supplementary Information).

### Thickness Optimization of gold and silicon layers

The optimized thickness of gold thin film and silicon nanosheet were obtained from the optimization of the sensitivity and FWHM. The SPR sensitivity serves as the key characteristic of SPR biosensor is defined in equation. (13) (See the Methods section). When the refractive index change of the biomolecules analyte layer (*∆n*_*bio*_ = 0.005) was fixed, the sensitivity was governed by the change in resonance angle that acquired before and after the adsorption of biomolecules on the surface of the MX_2_ layers surface. Upon investigation of the variation of change in the resonance angle as a function of the number of MX_2_ layers as shown in Figs 2d–f to 6d–f three main features were observed: (i) As the number of MX_2_ layers increased, the change in resonance angle increased until it reached a maximum followed by a rapid decreasing to quasi-zero. (ii) When the excitation wavelength and gold thickness were fixed, the reduced thickness of silicon nanosheet caused the peak of the change in resonance angle to shift toward a larger number of MX_2_ layers; similarly, if the excitation wavelength and the silicon thickness were fixed, the decreased thickness of gold thin film also led to a peak shift to a larger number of MX_2_ layers. (iii) With fixed thickness of silicon and gold, the redshifted excitation wavelengths induced the peak shift of change in resonance angle towards the larger number of MX_2_ layers. Similar behaviors were also observed in Figs 2a–c to 6a–c (i.e., at longer excitation wavelengths, a larger number of MX_2_ was required to achieve the minimum reflectivity dip). This showed that the angular sensitivity decreases with longer excited wavelengths^5^, thereby higher refractive index materials are required to enhance the evanescent field.

With optimized number of MX_2_ layers, the angular sensitivity and FWHM of the four silicon-MX_2_ enhanced models were then analyzed under five different excitation wavelengths. When the excitation wavelength was set at 600 nm, the highest sensitivity 155.68 Deg/RIU was achieved with optimum parameters of 35 nm thickness gold film, 7 nm thickness silicon and monolayer WS_2_. The FWHM was 17.4644 Deg with a minimum reflectivity of 2.5592 × 10^−2^ as shown in Table S1 (Supplementary Information). However, with the same thickness parameters and excitation wavelength, the highest sensitivity of silicon-MoSe_2_ enhanced model was only 104.56 Deg/RIU with a minimum reflectivity of 4.7627 × 10^−3^ as shown in Table S6 (Supplementary Information). This result can be explained by the different optical properties of WS_2_ and MoSe_2_. At 600 nm excitation wavelength, the real part of the dielectric constant of MoSe_2_ is 2 times as that of WS_2_, which indicates MoSe_2_ layers have higher energy absorption compared to WS_2_ layers. As a result, the *minR* of silicon-MoSe_2_ enhanced model is lower than that of silicon-WS_2_. However, the absorbed energy is not completely transferred to enhance the evanescent field due to the energy loss during the process. It is known that a dielectric material with a large real part and a small image part of the dielectric function have low energy loss, therefore the WS_2_ layers have much lower energy loss than that of MoSe_2_. Furthermore, the penetration depth of the evanescent field in the biomolecular analyte layer of the silicon-WS_2_ model is deeper than that of the silicon-MoSe_2_ model, since the real part of dielectric constant of WS_2_ is smaller than MoSe_2_ [6]. Consequently, the evanescent field of silicon-WS_2_ model is more sensitive than that of silicon-MoSe_2_ model to the refractive index change in biomolecular analyte. All these factors contributed to higher sensitivity in the silicon-WS_2_ enhanced scheme at 600 nm excitation wavelength. Similarly, the thickness combination for excitation wavelengths ranging from 633 nm to 1024 nm were also optimized (see Supplementary Information Tables S2–S20). Based on these results, we could conclude that although the MX_2_ layers with large real values of the dielectric constant contributed to the increased energy absorption, the intrinsic energy loss in these layers played a more significant role in the sensitivity of the multi-layered system.

### Influence of excitation wavelength

In order to obtain the best SPR sensing performance, it is also important to achieve a relatively low FWHM as it promises more accurate determination of the angular modulation. The value of the FWHM is mainly depends on two factors: excitation wavelength and the number of MX_2_ layers. As reported previously, using longer excitation wavelength results in a narrower resonance curve^6^. As for the latter factor, our simulation results showed that large number of MX_2_ layers generated a higher value of FWHM since the additional MX_2_ layers resulted in higher electron energy loss and reduced the accuracy. To investigate the effect of the excitation wavelength, we summarized the optimized thickness combinations of gold thin film and silicon nanosheet for five different excitation wavelengths. As shown in Table 1, three features were observed: (i) By using 600 nm, 633 nm, 660 nm and 785 nm excitation wavelengths, the best performances were all achieved in the silicon-WS_2_ sensing model except for the 1024 nm excitation wavelength which was attained in the silicon-MoS_2_ scheme. This was due to the hybrid effects of the energy absorption and energy loss of MX_2_ layers at the different excitation wavelengths. (ii) For the visible range, the optimized thickness of silicon was 7 nm and the WS_2_ layers were required to be ultra-thin (i.e., 1–3 layers), the predicted sensitivity were all above 140 Deg/RIU. However, for the near-infrared range (i.e., 785 nm and 1024 nm), the optimized thickness of silicon and gold were determined to be 5 nm and 40 nm respectively, with the optimized number of MX_2_ layers being at least 13 and above. (iii) It is worth noting that as the excitation wavelength redshifted, the optimized sensitivity decreased gradually. The dielectric constant changes significantly with the increasing incident wavelengths. The real part of the dielectric constant is related to the reflectivity of the interface while the imaginary part is indicative of the energy absorption. Therefore, according to Table 1, as the wavelengths progressed into the near-infrared region, the hybrid effects resulted in higher attenuation of the evanescent field and thus lower SPR sensitivity was observed^49^.

### Optimized scheme for each of the Silicon-MX_2_ model

**Figure 7:**
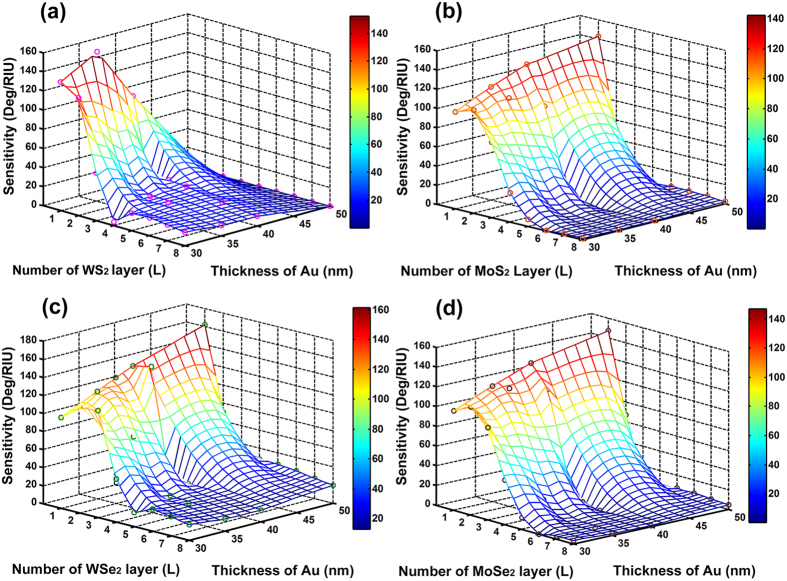
The variation of the sensitivity as a function of gold thin film thickness and number of MX_2_ layers with 7 nm thick silicon nanosheet. (**a**) The excitation wavelength is 600 nm in silicon-WS_2_ nanosheet enhanced model; (**b**) The excitation wavelength is 633 nm in silicon-MoS_2_ nanosheet enhanced model; (**c**) The excitation wavelength is 633 nm in silicon-WSe_2_ nanosheet enhanced model; (**d**) The excitation wavelength is 633 nm in silicon-MoSe_2_ nanosheet enhanced model.

**Figure 8:**
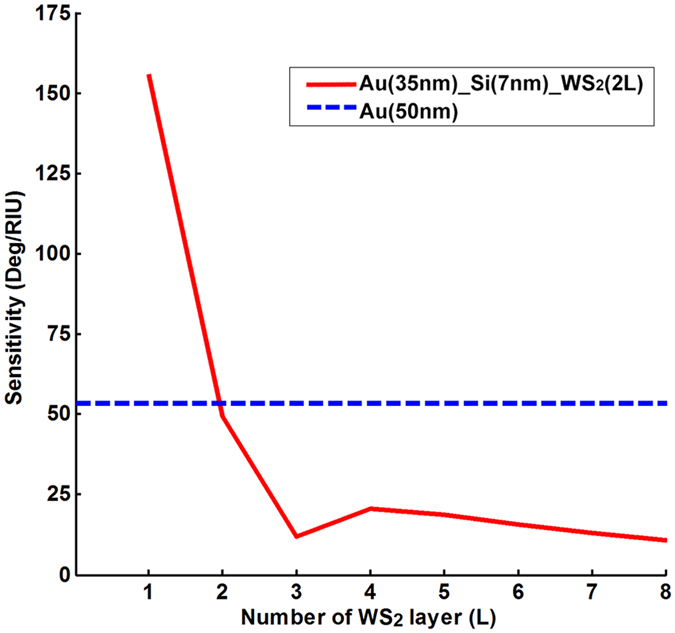
Comparison of the SPR sensing performances between the optimized scheme of 35 nm thick gold thin film, 7 nm thick silicon nanosheet and monolayer WS_2_ (the red solid line) and the conventional Kretschmann configuration with 50 nm thick Au substrate (the blue dashed line).

Finally, the parameters yielding the best performance for each of the silicon-WS_2_, silicon-WSe_2_, silicon-MoS_2_, silicon-MoSe_2_ enhanced SPR models are presented in Table 2. The optimized excitation wavelength and thickness of silicon nanosheet, gold thin film are the same for all the silicon-MX_2_ enhanced models except for the silicon-WS_2_ model. For the silicon-WS_2_ scheme, it possessed the highest sensitivity of 155.68 Deg/RIU with 35 nm thick gold and 7 nm silicon at the 600 nm wavelength. The 3D plots shown in Fig. 7a–d further validates the optimized parameters by depicting the sensitivity as a function of number of MX_2_ layers and gold thickness with the corresponding optimized silicon thickness and excitation wavelengths. From Fig. 7b–d, the highest value of sensitivity seemed to be attainable with 50 nm thick gold thin film and monolayer MX_2_. However, the minimum SPR reflectivity *minR* under these condition were greater than 0.03, leading to low energy-transfer efficiencies. In addition, the slopes of the sensitivity as a function of MX_2_ layers coated on 40 nm-thickness Au thin film were the sharpest among all the other Au thicknesses. Therefore, we concluded that the highest sensitivity of silicon-MoS_2_, silicon-MoSe_2_ and silicon-WSe_2_ models were obtained with 40 nm thick gold films. We also compared the sensitivity of our optimized configuration with the well-known conventional Kretschmann configuration (with 50 nm thickness gold thin film). As shown in Fig. 8, the sensitivity of our optimized silicon-WS_2_ enhanced configuration (155.68 Deg/RIU as shown in red solid line) is 3 times more sensitive than that of the Kretschmann design (53.40 Deg/RIU as shown in blue dashed line). For the Kretschmann configuration without any dielectric layers, the only absorbing medium in the system is the metal film; whereas in the silicon-WS_2_ enhanced structure the additional silicon nanosheet and MX_2_ layers can serve as the absorption medium as well. Moreover, the high refractive index of silicon nanosheet and the high real part of MX_2_ dielectric constant can enhance the evanescent field at the metal interface^49^. These double effects result in drastic sensitivity enhancement in the silicon-WS_2_ enhanced structure. In order to demonstrate the validity of our N-layer 2D models, we have experimentally tested the SPR sensing ability with 3-layer graphene-coated Au thin film. And both the angular and phase measurement results matched well with our theoretical analyses (see Supplementary Figs S1–S3).

## Conclusion

In this study, we demonstrated a silicon nanosheet and 2D MX_2_ enhanced surface plasmon resonance biosensor. Based on the Kretschmann configuration, the system consists of SF10 triangular prism, gold thin film, silicon nanosheet and 2D MX_2_ film (MoS_2_/MoSe_2_/WS_2_/WSe_2_). To investigate the enhancement effect of each MX_2_ materials, we designed four enhanced models, namely, silicon-WS_2_, silicon-WSe_2_, silicon-MoS_2_ and silicon-MoSe_2_. Maxwell’s equations, Fresnel equations and transfer matrix method were used to analyze the change in resonance angle and the corresponding sensitivity for angular modulation. To study the influence of the excitation wavelengths to sensing performance, we studied five different excitation wavelengths, namely, 600 nm, 633 nm, 660 nm, 785 nm and 1024 nm. The results showed that the silicon nanosheet together with each of the four types of 2D MX_2_ layers could compensate the SPR effect of gold and significantly improve the sensitivity of the biosensor. However, excessive MX_2_ layers would result in increased energy loss and reduced the sensitivity. Therefore, in order to optimize the sensitivity, the thickness of gold film, silicon nanosheet and MX_2_ layers were investigated to minimize the reflectance and width of SPR curve to achieve a system with higher angular scanning accuracy. The combination of the optimized parameters for each excitation wavelength and each silicon-MX_2_ enhanced model were also presented with the highest SPR sensitivity of 155.68 Deg/RIU achieved using 35 nm thick gold film, 7 nm thick silicon nanosheet and a monolayer WS_2_ under the illumination of a 600 nm excitation wavelength source.

## Methods

### The wavelength-dependent refractive index for each layer

In this study, the theoretical analyses were performed with excitation light wavelengths in visible and near infrared ranges. Five wavelengths were chosen as 600 nm, 633 nm, 660 nm, 785 nm and 1024 nm respectively. The proposed configuration consists of 6 layer components, namley the SF10 prism, gold thin film, silicon nanosheet, 2D MX_2_ nanolayers (MoS_2_/WS_2_/MoSe_2_/WSe_2_), biomolecular analyte layer and sensing medium layer. The first component is the SF10 prism whose refractive index is given by^50^:




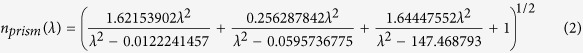




where *λ* represents the wavelength of the light source in μm and Eq. (2) is valid for wavelengths from 0.38 μm to 2.5 μm. The refractive index of the gold thin film is determined using the Drude model by:




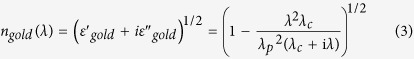




where *λ*_*p*_ (1.6826 × 10^−7^ m) denotes the plasma wavelength and *λ*_*c*_ (8.9342 × 10^−6^ m) is the collision wavelength. *λ* in Eq. (3) represents the wavelength of the light source in μm, which is valid from 0.18 μm to 1.94 μm^51^,^52^,^53^. The thickness of the gold thin film was varied from 30 nm to 50 nm in this study. The refractive index of the silicon nanosheet is calculated by:




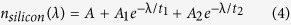




where *A* = 3.44904, *A*_*1*_ = 2271.88813, *A*_*2*_ = 3.39538, *t*_*1*_ = 0.058304 and *t*_*2*_ = 0.30384, and *λ* is the wavelength in μm^54^. The thickness of the silicon sheet was varied from 0 nm to 7 nm. The fourth layer is the core functional layer −2D MX_2_ (MoS_2_/WS_2_/MoSe_2_/WSe_2_) whose refractive indexs and the monolayer thickness *t*_*MX2*_ are summarised in Table 3 55,56. The thickness of MX_2_ layers *d*_*MX2*_ is described by *d*_*MX2*_ = *t*_*MX2*_ × *L*, where *L* represents the number of MX_2_ layers. The fifth layer is the biomolecular analyte that dissolved in sensing medium. The change in refractive index of this layer can be induced by absorption of the biomolecules on the MX_2_ surface. Here the refractive index of this layer can be represented as *n*_*analyte*_ = 1.330 + *∆n*_*bio*_, where *∆n*_*bio*_ indicates the change in refractive index of the biomolecular analyte. The thickness of this layer *d*_*analyte*_ is fixed at 100 nm. The last layer is the sensing medium, the refractive index is defined as *n*_*solvent*_ = 1.330.

### Reflection coefficient (*r*_*p*_) and Reflectivity (*R*_*p*_)

In this study, the SPR biosensor model is based on the well-known Kretchmann’s attenuated total reflection (ATR) structure^7^. The incident light passes through the prism and is totally reflected at the base of the prism. Most of the light energy is absorbed by the metal and dielectric layers to generate the evanescent wave which propagates along the interface and penetrates into the sensing film. When the evanescent wave vector *k*_*x*_ matches with the surface plasmon wave vector *k*_*sp*_, the resonance occurs as shown in Eq. (1). In order to analyse our N-layer structure model, the transfer matrix method (TMM) was employed. Each layer in our system was stacked horizontally in z-axis direction. All layers are defined by the parameters *n*_*k*_ , *ε*_*k*_ and *d*_*k*_ , which represent the refractive index, dielectric constant and thickness of the *k*_th_ layer, respectively. *θ*_*k*_ denotes the incident angle of the *k*_th_ layer and *λ* stands for the excitation wavelength.

To obtain the reflected intensity, Fresnel equations and relevant boundary conditions were introduced. In the calculations, the first boundary of the tangential fields was assumed as *Z*_*1*_ = *0*, and the tangential fields at the last boundary *Z*_*N−1*_, there by giving rise to Eq. (5) as follows:




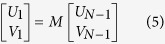




where *U*_*1*_, *U*_*N−1*_ represent the tangential components of the first and the last layers in the electric fields, while *V*_*1*_, *V*_*N−1*_ denote the corresponding components in magnetic fields. *M* refers to the characteristic matrix of the *N*-layer model. For the *p*-polarized light, the characteristics matrix is given by:




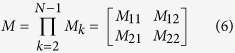




With




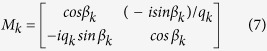




Where




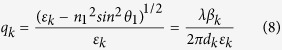







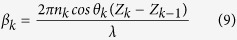




Thus, the four elements *M*_*11*_, *M*_*12*_, *M*_*21*_and *M*_*22*_ of the matrix *M* can be calculated. According to the Fresnel’s equations, the complex reflection coefficient *r*_*p*_ of *p*-polarized incident electromagnetic field can be described by:




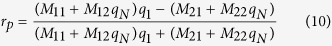




where, *q*_*1*_and *q*_*N*_ can be calculated from Eq. (8), which represent the relative components of the first layer and the *N*_th_ layer respectively. Therefore, the system reflectivity *R*_*p*_ for the *p*-polarized incident light can then be obtained by taking the square of the reflection coefficient *r*_*p*_, shown as follows:




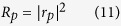




The angular sensitivity is defined as the ratio between the change of the resonance angle to the change of the analyte refractive index^5^ in Eq. (12).




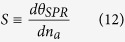




It can be simplified to




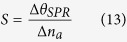




The sensitivity describes the change in the optical signal (i.e., changes in the resonance angle) corresponding to the minute changes in the refractive index of the biomolecular analyte. The sensitivity depends on the evanescent field strength which directly related to the absorbed light energy. Another key parameter of interest is the width of the SPR curve, which related to the accuracy of the sensing system. The width of SPR curve is determined by the dielectric function of the metallic silicon-MX_2_ thin film. Generally, a large value of 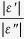. sults in a narrow resonance curve^6^. In this work, the full width at half maximum (FWHM) is calculated to investigate the SPR curve width, as shown in Eq. (14),




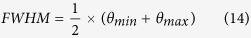




where *θ*_*min*_ is the incident angle corresponding to the minimum reflected intensity, and *θ*_*max*_ is the incident angle with the maximum reflected intensity base on the SPR curve.

## Additional Information

**How to cite this article**: Ouyang, Q. *et al.* Sensitivity Enhancement of Transition Metal Dichalcogenides/Silicon Nanostructure-based Surface Plasmon Resonance Biosensor. *Sci. Rep.* **6**, 28190; doi: 10.1038/srep28190 (2016).

## Supplementary Material

Supplementary Information

## Figures and Tables

**Figure 1:**
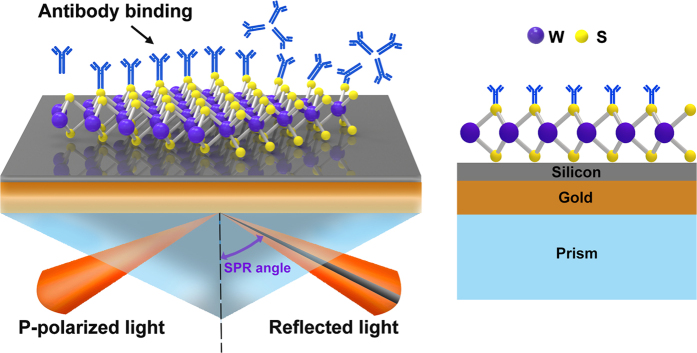
Schematic diagram of silicon-WS_2_/ nanosheets-enhanced surface plasmon resonance biosensor.

**Table 1:** The optimized values of gold thin film, silicon nanosheet thickness and the number of MX_2_ layers with corresponding changes in resonance angle, sensitivities and FWHMs in SPR curves for 600 nm, 633 nm, 660 nm, 785 nm and 1024 nm excitation wavelengths.

Excitation wavelength (nm)	Type of TMDC	Gold thickness (nm)	Silicon thickness (nm)	Number of WS_2_ layers (*L*)	Minimum Reflectivity	*Δθ*_*SPR*_(Deg) (*Δn*_*bio*_ = 0.005)	Sensitivity (Deg/RIU)	FWHM (Deg)
600	WS_2_	35	7	1	2.5592 × 10^−2^	0.7784	155.68	17.4644
633	WS_2_	40	7	1	2.4099 × 10^−2^	0.7394	147.88	16.2417
660	WS_2_	40	7	3	3.3778 × 10^−5^	0.7282	145.64	16.8245
785	WS_2_	40	5	13	2.5767 × 10^−3^	0.6395	127.90	15.5232
1024	MoS_2_	40	5	26	1.6573 × 10^−6^	0.4499	89.98	17.5445

**Table 2:** The optimized values of gold thin film, silicon nanosheet thickness and the number of MX_2_ layers with corresponding excitation wavelength, change in resonance angle, sensitivity and FWHM of the SPR curve for each silicon-MX_2_ nanosheet enhanced model.

Excitation wavelength (nm)	Type of TMDC	Gold thickness (nm)	Silicon thickness (nm)	Number of WS_2_ layers (*L*)	Minimum Reflectivity	*Δθ*_*SPR*_ (Deg) (*Δn*_*bio*_ = 0.005)	Sensitivity (Deg/RIU)	FWHM (Deg)
600	WS_2_	35	7	1	2.5592 × 10^−2^	0.7784	155.68	17.4644
633	MoS_2_	40	7	1	1.1513 × 10^−5^	0.6586	131.70	17.5728
633	WSe_2_	40	7	2	5.3807 × 10^−3^	0.7070	141.40	17.2340
633	MoSe_2_	40	7	1	2.0438 × 10^−3^	0.6584	131.68	17.0915

**Table 3:** The optical constants of 2D MoS_2_, MoSe_2_, WS_2_, and WSe_2_ with different excitation wavelengths from 600 nm to 1024 nm.

TMDC	*T*_*MX2*_ (nm)	*λ* = 600 nm	*λ* = 633 nm	*λ* = 660 nm	*λ* = 785 nm	*λ* = 1024 nm
MoS_2_	0.65	*ɛ*′ = 17.7967,	*ɛ*′ = 24.4368,	*ɛ*′ = 23.4129,	*ɛ*′ = 21.4675,	*ɛ*′ = 20.8372,
		*ɛ″* = 10.7801	*ɛ″* = 11.9121	*ɛ″* = 12.5610	*ɛ″* = 1.0781	*ɛ″* = 0.9662
MoSe_2_	0.70	*ɛ*′ = 21.3204,	*ɛ*′ = 20.3560,	*ɛ*′ = 19.3366,	*ɛ*′ = 17.7994,	*ɛ*′ = 14.9028,
		*ɛ″* = 10.9486	*ɛ″* = 9.3039	*ɛ″* = 8.4366	*ɛ″* = 7.0709	*ɛ″* = 2.7611,
WS_2_	0.80	*ɛ*′ = 12.0258,	*ɛ*′ = 23.8511,	*ɛ*′ = 19.9701,	*ɛ*′ = 16.0968,	*ɛ*′ = 25.0445,
		*ɛ″* = 4.2578	*ɛ″* = 3.0578	*ɛ″* = 1.8420	*ɛ″* = 0.3203	*ɛ″* = 2.5676,
WSe_2_	0.70	*ɛ*′ = 19.4125,	*ɛ*′ = 20.5156,	*ɛ*′ = 18.7344,	*ɛ*′ = 19.0563,	*ɛ*′ = 23.6474,
		*ɛ″* = 8.4135	*ɛ″* = 3.9423	*ɛ″* = 2.1955	*ɛ″* = 0.3205	*ɛ″* = 2.3801

The dielectric constant is described by *ε* = *ε*′ + *ε*″*i*, where the real part *ε*′ relates to the stored energy within the medium and the imaginary part *ε*″ relates to the dissipation of energy within the medium. (The complex refractive index 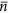 is defined as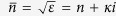, where the real part *n* indicates the phase velocity, while the imaginary part *κ* known as the extinction coefficient refers to the mass attenuation coefficient).
