# Supplementary material for: Sensitivity Enhancement of Transition Metal Dichalcogenides/Silicon Nanostructure-based Surface Plasmon Resonance Biosensor
Source: Sci Rep. 2016 Jun 16;6:28190. doi: 10.1038/srep28190 (PMC4910054; doi:10.1038/srep28190)
Supplement: Supplementary Information [file srep28190-s1.pdf]

Supplementary Information

Sensitivity Enhancement of Transition Metal Dichalcogenides/Silicon Nanostructure-based Surface Plasmon Resonance Biosensor

Qingling Ouyang[1,2†], Shuwen Zeng[1,2*†], Li Jiang[2,5], Liying Hong[1], Gaixia Xu[2,4], Xuan-Quyen Dinh[2], Jun Qian[5], Sailing He[5], Junle Qu[4*], Philippe Coquet[2,3] and Ken-Tye Yong[1,2*]

[1]School of Electrical and Electronic Engineering, Nanyang Technological University, Singapore, 639798

[2]CINTRA CNRS/NTU/THALES, UMI 3288, Research Techno Plaza, 50 Nanyang Drive, Border X Block, Singapore, 637553

[3]Institut d'Electronique, de Microélectronique et de Nanotechnologie (IEMN), CNRS UMR 8520 – Université de Lille 1, 59650 Villeneuve d'Ascq, France

[4]Key Laboratory of Optoelectronics Devices and Systems of Ministry of Education/Guangdong Province, College of Optoelectronic Engineering, Shenzhen University, Shenzhen, P. R. China

[5]State Key Laboratory of Modern Optical Instrumentation, Centre for Optical and Electromagnetics Research, Zhejiang University, Hangzhou 310058, China

[†] These authors contributed equally to this work

*Correspondence author: E-mail: swzeng@ntu.edu.sg; jlqu@szu.edu.cn; ktyong@ntu.edu.sg

1. Experiment

1.1. Material

Three-layer graphene was grown on high-purity (99.99%) copper foil by use of low-pressure chemical vapor deposition as the one used in our previous work [*Zeng et al., Adv. Mater. 27, 6163, (2015)*]. Glycerin (99%) was purchased from Sigma-Aldrich. Ultrapure deionized (DI) water was obtained by Spectra-Teknik water purification system.

1.2. SPR Setup

One phase-sensitive SPR setup (Fig. S1a) was employed for the experimental investigation of phase. A 4mW 632.8nm He-Ne laser with a beam spot size of 1mm was used to excite the surface electromagnetic waves. P-polarized light and s-polarized light were splitted by a polarized beam splitter. In the beam of p-polarized light, the configuration was based on the well-known Krestchmann configuration including a right-angle BK7 coupling prism with 40 mm length of legs which was immobilized on a rotation stage. The thin Au film graphene stacks with a flow chamber was attached to the face of prism by optical matching oil (Cargille Labs). Sample solutions could be injected into the flow chamber by a syringe pump to interact with the sensing film. The incident p-polarized light passed through the sensing film and then reflected out. In the beam of s-polarized light, a galvo-mirror (Thorlabs, GVS001) was driven by sine wave signals oscillating at 86 Hz to generate optical path difference and obtain complete sine waves for the interference light of p-polarized light and s-polarized light. The intensity of final interfered light was detected by a photo detector and collected by a data acquisition card (NI PCI-6115) using a Labview program. A point-wise arcsine algorithm was used to extract the phase difference between p-polarized light and s-polarized light.

Another angle-sensitive SPR setup (Fig. S1b) was used for the measurement of reflectivity of the thin Au film graphene stacks. The incident light was set as the p-polarized light by a polarizer. The light beam was focused on the homogeneous part of the graphene stacks on the Au film attached to the hypotenuse face of the prism and then reflected out. The incident angle could be changed by rotating the rotation stage. The intensity of the reflected light was changing with the incident angle, which could be monitored by a high-precision optical power meter (Newport 2832C). Then we could get the reflectance for each incident angle.

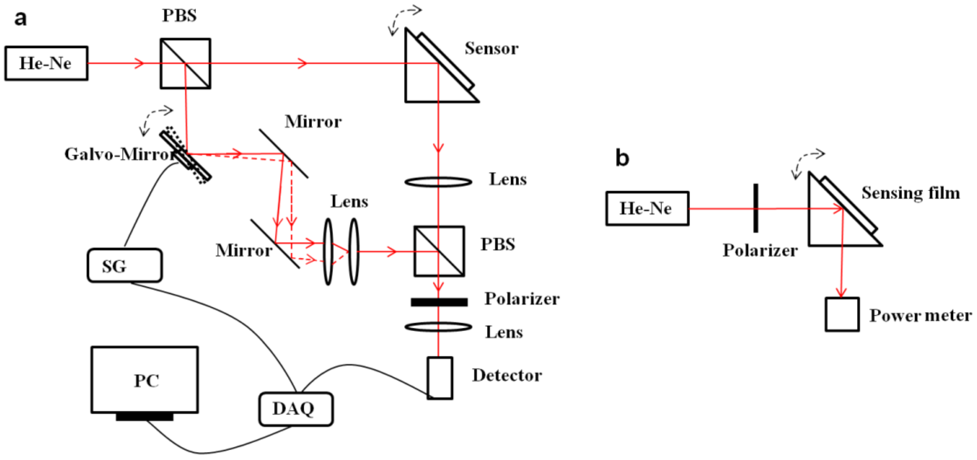

Figure S1. Schematic diagram showing the setup of (**a**) phase-sensitive SPR setup and (**b**) angle-sensitive SPR setup.

1.3. Result

Reflectance and differential phase were measured for both bare Au sensing film and 3-layer graphene-coated Au sensing film. In the measurement of phase sensitivity of bare Au sensing film and 3-layer graphene-coated Au sensing film, glycerin solutions (Sigma-Aldrich) with different weight ratios were injected into the flow chambers. The corresponding refractive index from pure DI water to 10% glycerin solution was measured to be from 1.332 to 1.344 by an abbe refractometer (2WAJ).

For 50nm bare Au sensing film with 2.5 nm titanium adhesion layer, experimental data of reflectance in the air with respect to different incident angle were measured using angle-sensitive SPR setup, where the blue line was fitted for eye guide (Fig. S2a). The measured resonance angle was 36.2º, which could well match the simulated data by solving the Fresnel's equation, estimated resonance angle was 36.1º, (Fig. S2b). Change in differential phase of different weight ratios of glycerin solutions was obtained by phase-sensitive SPR setup, where the blue line fitted the experimental data (Fig. S2c). The differential phase was linearly changed from DI water (1.332) to 1.5% glycerin solutions (1.3338), corresponding to a phase sensitivity of 16,944 Deg/RIU, which was in good consistent with the simulation result (Fig. S2d).

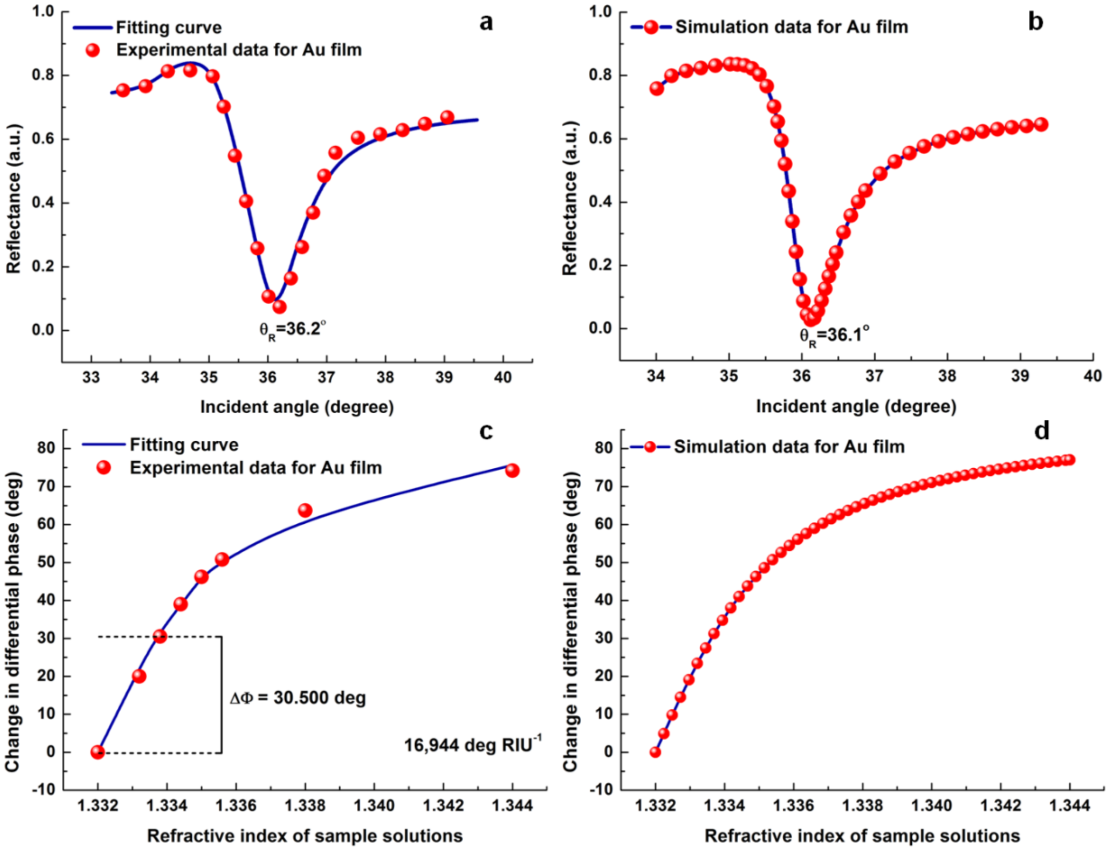

Figure S2. The thickness of the bare Au sensing film is 50nm with 2.5 nm titanium adhesion layer. Variation of reflectance in the air, (**a**) experimental result and (**b**) simulation result, with respect to angle of incidence; Change in differential phase, (**c**) experimental result and (**d**)

simulation result, for various weight ratios of glycerin solutions.

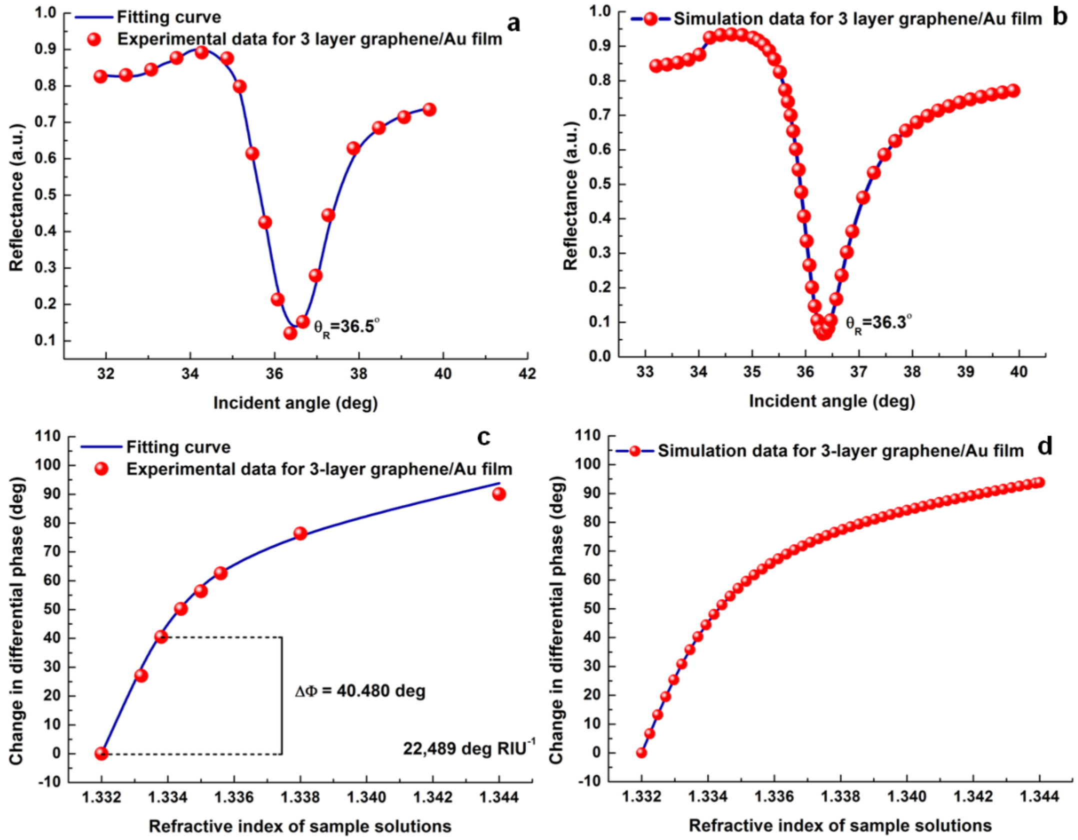

Figure S3. Three-layer graphene was coated on the 50 nm bare Au sensing film with 2.5 nm titanium adhesion layer. Variation of reflectance in the air, (**a**) experimental result and (**b**) simulation result, with respect to angle of incidence; Change in differential phase, (**c**) experimental result and (**d**) simulation result, for various weight ratios of glycerin solutions.

For 3-layer graphene-coated Au sensing film, the reflectance in the air was measured as well and the obtained resonance angle was 36.5º (Fig. S3a) in good agreement with the simulation result (estimated resonance angle 36.3º) (Fig. S3b). A phase sensitivity of 22,489 Deg/RIU could be calculated corresponding to the linearly phase change from DI water to 1.5% glycerin solutions (Fig. S3c), matching well with the result of simulation (Fig. S3d). Both the reflectance and differential phase change were in good consistent with the simulation result for bare Au sensing film and 3-layer graphene-coated Au sensing film. The phase sensitivity of 3-layer graphene-

coated Au sensing film was 1.33 times higher than the one of bare Au sensing film, which could validate that 3-layer graphene could enhance the performance of whole sensing structure.

2. Theoretical analysis

2.1. Angular interrogation

A silicon nanosheet and 2D MX_2 heterostructure based surface plasmon resonance biosensor configuration is proposed. The four enhanced models, namely, silicon-WS_2, silicon-WSe_2, silicon-MoS_2 and silicon-$MoSe_2$ were analyzed individually through the transfer matrix method. In order to study the angular sensitivity, the reflectivity, resonance angle shift as well as the FWHM of all the four silicon-MX_2 models were studied under five excitation wavelengths (600 nm, 633 nm, 660 nm, 785 nm and 1024 nm), as shown in Table S1-20. Therefore, the optimized parameters of each of the silicon-MX_2 enhanced models were obtained. Here, we plot the SPR curves before (the blue line) and after (the red line) the adsorption of the ssDNA with a refractive index change at 0.005, as shown in Fig. S4-S7. Specifically, in silicon-WS_2 enhanced model, with the optimized parameters of 35 nm gold, 7 nm silicon nanosheet and monolayer WS_2 and 600 nm excitation wavelength, the resonance angle (SPR angle) shift can reach to a maximum of 0.7786°, as shown in Fig. S4. In silicon-MoS_2 and silicon-$MoSe_2$ enhanced models, the optimized parameters were the same: 40 nm thick gold thin film, 7 nm silicon and monolayer WS_2/$MoSe_2$ at 633 nm excitation wavelength. The resonance angle shift were 0.6586° and 0.6854° respectively, see Fig. S5 and Fig. S6. At 633 nm excitation wavelength, the optimized thickness parameters of the silicon-WSe_2 enhanced model were 40 nm gold, 7 nm silicon and bilayer WSe_2, the resonance angle change reached to 0.7070°, as shown in Fig. S7. In the

theoretical analysis, we considered the refractive index of 2D MX_2 materials is independent on the thickness in nanoscale range. Our theoretical studies were based on the experimental conditions and parameters. The multi-layer 2D MX_2 were fabricated through stacking each single 2D layer on the sensing substrate. Thus, the refractive index of each layer of the N-layered MX_2 thin film was consistent in the theoretical analysis.

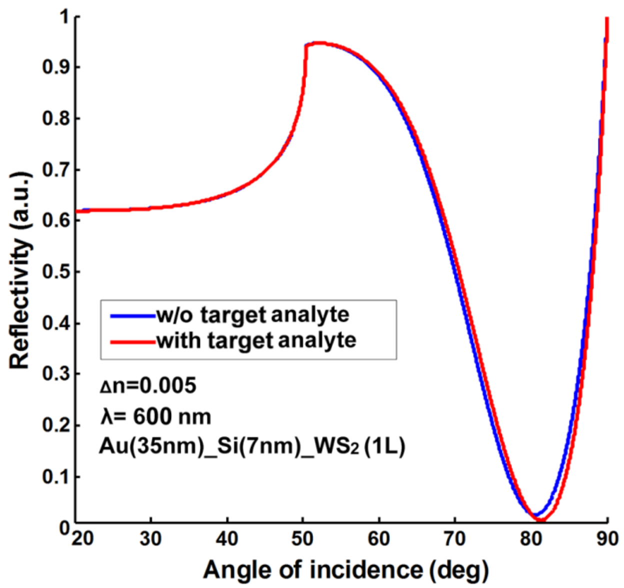

Figure S4. The reflectivity as a function of the incident angle before (blue line) and after (red line) the adsorption of the target analyte at 600 nm excitation wavelength under the optimized parameters of 35 nm gold thin film, 7 nm silicon nanosheet and monolayer WS_2.

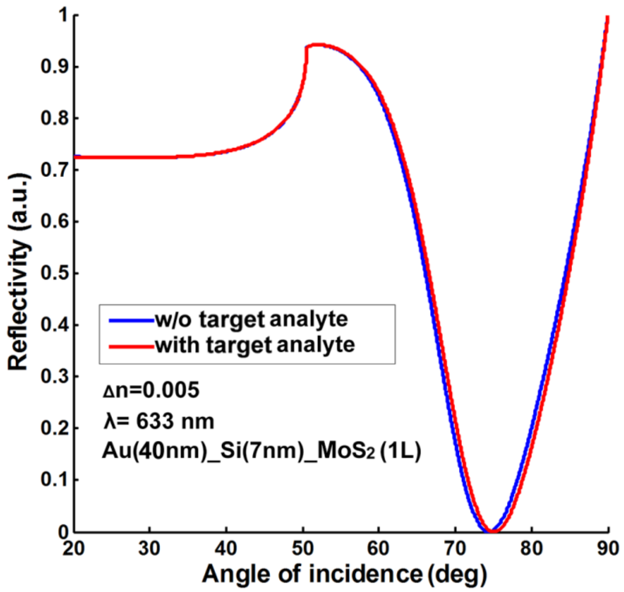

Figure S5. The reflectivity as a function of the incident angle before (blue line) and after (red line) the adsorption of the target analyte at 633 nm excitation wavelength under the optimized parameters of 40 nm gold thin film, 7 nm silicon nanosheet and monolayer MoS_2.

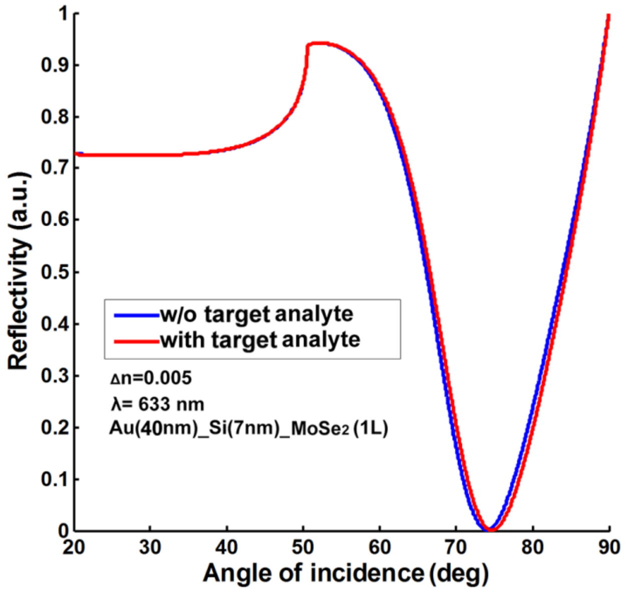

Figure S6. The reflectivity as a function of the incident angle before (blue line) and after (red line) the adsorption of the target analyte at 633 nm excitation wavelength under the optimized parameters of 40 nm gold thin film, 7 nm silicon nanosheet and monolayer $MoSe_2$.

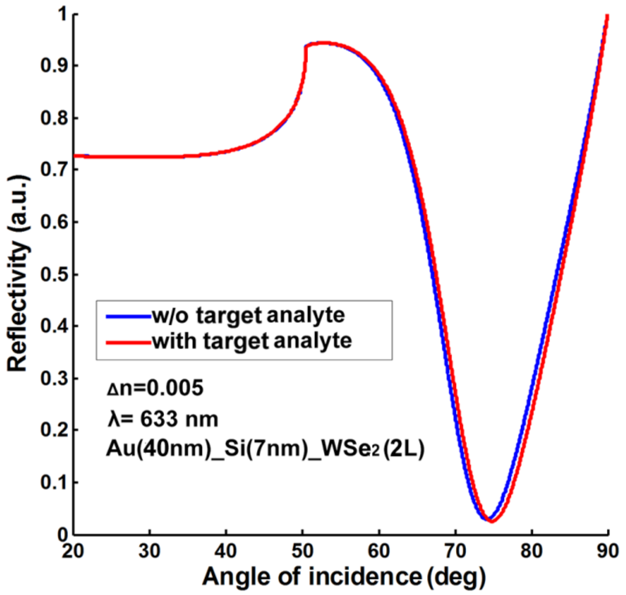

Figure S7. The reflectivity as a function of the incident angle before (blue line) and after (red line) the adsorption of the target analyte at 633 nm excitation wavelength under the optimized parameters of 40 nm gold thin film, 7 nm silicon nanosheet and bilayer WSe_2.

Table S1

The optimized values of gold thin film, silicon nanosheet thickness and the number of WS_2 layers with corresponding change in resonance angle and FWHM in SPR curve for 600nm excitation wavelength.

Au thickness (nm)	Si thickness (nm)	Number of WS_2 layers (L)	Minimum Reflectivity	θ_{SPR} w/o biomolecules (Deg)	θ_{SPR} with biomolecules (Deg)	$\Delta\theta_{SPR}$ (Δn_{bio}=0.005) (Deg)	Sensitivity (Deg/RIU)	FWHM (Deg)
30	0	9	1.0306×10^{-3}	68.6670	69.1079	0.4409	88.18	22.3953
35	0	6	4.5593×10^{-4}	63.4245	63.8173	0.3928	78.56	15.3217
40	0	4	9.6791×10^{-4}	60.6633	61.0124	0.3491	69.82	9.0687
50	0	0	6.0974×10^{-5}	56.7966	57.0636	0.2670	53.40	2.3134
30	5	5	4.4617×10^{-4}	79.2675	79.7532	0.4857	97.14	21.3792
35	5	3	7.4642×10^{-3}	74.8978	75.5304	0.6326	126.52	19.5973
40	5	2	2.3410×10^{-4}	72.6477	73.2982	0.6505	130.10	16.7183
50	5	0	2.6443×10^{-3}	67.5897	68.1553	0.5656	113.12	8.0491
30	7	3	7.7163×10^{-3}	83.6202	83.8693	0.2491	49.82	18.0984
35	7	1	2.5592×10^{-2}	80.5633	81.3417	0.7784	155.68	17.4644

Table S2

The optimized values of gold thin film, silicon nanosheet thickness and the number of WS_2 layers with corresponding change in resonance angle and FWHM in SPR curve for 633nm excitation wavelength.

Au thickness (nm)	Si thickness (nm)	Number of WS_2 layers (L)	Minimum Reflectivity	θ_{SPR} w/o biomolecules (Deg)	θ_{SPR} with biomolecules (Deg)	$\Delta\theta_{SPR}$ (Δn_{bio}=0.005) (Deg)	Sensitivity (Deg/RIU)	FWHM (Deg)
30	0	9	2.3193×10^{-3}	81.5711	81.8895	0.3184	63.68	20.9457
35	0	7	4.4970×10^{-3}	73.2828	73.8314	0.5486	109.72	20.9152
40	0	5	8.3104×10^{-4}	65.3435	65.7847	0.4412	88.24	14.0670
50	0	0	1.4822×10^{-5}	56.1871	56.4280	0.2409	48.18	2.1004
30	5	5	2.5504×10^{-2}	84.4157	84.5338	0.1181	23.62	17.4124
35	5	4	1.3915×10^{-2}	82.1903	82.5787	0.3884	77.68	18.1349
40	5	3	2.6591×10^{-3}	77.5365	78.2352	0.6987	139.74	17.9590
50	5	0	5.0702×10^{-4}	63.7243	64.1544	0.4301	86.02	5.9887
30	7	3	1.0791×10^{-3}	84.8480	85.1460	0.2980	59.60	16.3374
35	7	2	1.1042×10^{-2}	81.6272	82.2923	0.6651	133.02	17.3960
40	7	1	2.4099×10^{-2}	75.8606	76.6000	0.7394	147.88	16.2417
50	7	0	5.5667×10^{-3}	70.7232	71.3733	0.6501	130.02	10.1617

Table S3
The optimized values of gold thin film, silicon nanosheet thickness and the number of WS_2 layers with corresponding change in resonance angle and FWHM in SPR curve for 660nm excitation wavelength.

Au thickness (nm)	Si thickness (nm)	Number of WS_2 layers (L)	Minimum Reflectivity	θ_{SPR} w/o biomolecules (Deg)	θ_{SPR} with biomolecules (Deg)	$\Delta\theta_{SPR}$ (Δn_{bio}=0.005) (Deg)	Sensitivity (Deg/RIU)	FWHM (Deg)
30	0	12	6.9049×10^{-3}	83.6359	83.8531	0.2172	43.44	18.5826
35	0	10	3.2174×10^{-3}	77.4934	78.0683	0.5749	114.98	20.2094
40	0	8	6.0830×10^{-4}	69.6006	70.1215	0.5209	104.18	16.8688
50	0	0	2.9483×10^{-4}	55.7750	55.9976	0.2226	44.52	1.9505
30	5	7	2.9289×10^{-3}	84.9508	85.1453	0.1945	38.90	16.4019
35	5	6	1.0379×10^{-2}	83.0225	83.3793	0.3568	71.36	17.2624
40	5	5	7.9432×10^{-3}	79.5416	80.1900	0.6484	129.68	17.5843
50	5	0	4.7825×10^{-4}	61.7097	62.0757	0.3660	73.20	4.9425
30	7	5	1.9914×10^{-4}	85.5051	85.7139	0.2088	41.76	15.3163
35	7	4	1.7959×10^{-3}	83.6110	84.0177	0.4067	81.34	16.3604
40	7	3	3.3778×10^{-5}	79.7827	80.5109	0.7282	145.64	16.8245
50	7	0	1.4158×10^{-3}	66.5248	67.0197	0.4949	98.98	7.8452

Table S4
The optimized values of gold thin film, silicon nanosheet thickness and the number of WS_2 layers with corresponding change in resonance angle and FWHM in SPR curve for 785nm excitation wavelength.

Au thickness (nm)	Si thickness (nm)	Number of WS_2 layers (L)	Minimum Reflectivity	θ_{SPR} w/o biomolecules (Deg)	θ_{SPR} with biomolecules (Deg)	$\Delta\theta_{SPR}$ (Δn_{bio}=0.005) (Deg)	Sensitivity (Deg/RIU)	FWHM (Deg)
35	0	20	8.2664×10^{-4}	85.1560	85.5004	0.3444	68.88	14.3217
40	0	19	3.5797×10^{-3}	83.0847	83.5818	0.4971	99.42	15.2830
50	0	0	1.0244×10^{-2}	54.4952	54.6566	0.1614	32.28	1.4493
30	5	16	4.6561×10^{-4}	87.3434	87.4381	0.0947	18.94	11.0503
35	5	14	2.4740×10^{-2}	84.3228	84.8311	0.5083	101.66	14.7978
40	5	13	2.5767×10^{-3}	81.9147	82.5542	0.6395	127.90	15.5232
50	5	0	8.3559×10^{-3}	57.1765	57.3998	0.2233	44.66	2.6852
30	7	14	2.2110×10^{-2}	87.5234	87.5085	0.0149	2.98	10.4471
35	7	12	9.2000×10^{-3}	85.1560	85.5773	0.4213	84.26	13.9815
40	7	11	6.7116×10^{-5}	82.9231	83.5107	0.5876	117.52	14.9616
50	7	0	7.3315×10^{-3}	58.8242	59.0838	0.2596	51.92	3.5672

Table S5

The optimized values of gold thin film, silicon nanosheet thickness and the number of WS_2 layers with corresponding change in resonance angle and FWHM in SPR curve for 1024nm excitation wavelength.

Au thickness (nm)	Si thickness (nm)	Number of WS_2 layers (L)	Minimum Reflectivity	θ_{SPR} w/o biomolecules (Deg)	θ_{SPR} with biomolecules (Deg)	$\Delta\theta_{SPR}$ (Δn_{bio}=0.005) (Deg)	Sensitivity (Deg/RIU)	FWHM (Deg)
30	0	26	2.9561×10^{-3}	84.5610	84.7037	0.1427	28.54	17.6854
35	0	23	8.4630×10^{-5}	79.3182	79.6444	0.3262	65.24	20.7662
40	0	4	2.3986×10^{-5}	54.5510	54.6765	0.1255	25.10	2.5545
30	5	21	1.7205×10^{-3}	84.0361	84.2409	0.2048	40.96	17.8724
35	5	19	1.3643×10^{-5}	80.3472	80.6762	0.3290	65.80	19.7136
40	5	4	3.2235×10^{-5}	56.2789	56.4394	0.1605	32.10	4.1127
30	7	20	3.9117×10^{-3}	85.0778	85.2133	0.1355	27.10	16.4240
35	7	17	1.0331×10^{-3}	79.5564	79.9109	0.3545	70.90	19.7501
40	7	4	1.3462×10^{-5}	57.2099	57.3881	0.1782	35.64	5.0626

Table S6

The optimized values of gold thin film, silicon nanosheet thickness and the number of $MoSe_2$ layers with corresponding change in resonance angle and FWHM in SPR curve for 600nm excitation wavelength.

Au thickness (nm)	Si thickness (nm)	Number of $MoSe_2$ layers (L)	Minimum Reflectivity	θ_{SPR} w/o biomolecules (Deg)	θ_{SPR} with biomolecules (Deg)	$\Delta\theta_{SPR}$ (Δn_{bio}=0.005) (Deg)	Sensitivity (Deg/RIU)	FWHM (Deg)
30	0	5	5.5708×10^{-4}	64.6016	64.9844	0.3828	76.56	21.8215
35	0	3	1.1107×10^{-3}	60.7113	61.0508	0.3395	67.90	13.1344
40	0	2	7.7992×10^{-4}	59.1615	59.4776	0.3161	63.22	7.9253
50	0	0	6.0974×10^{-5}	56.7966	57.0636	0.2670	53.40	2.3134
30	5	3	2.5544×10^{-4}	76.6972	77.1832	0.4860	97.20	22.8996
35	5	2	2.8247×10^{-5}	74.1700	74.7548	0.5848	116.96	20.6998
40	5	1	7.6602×10^{-4}	70.8050	71.4040	0.5990	119.80	16.1867
50	5	0	2.6443×10^{-3}	67.5897	68.1553	0.5656	113.12	8.0491
30	7	2	1.4748×10^{-2}	82.3111	82.5772	0.2661	53.22	19.8431
35	7	1	4.7627×10^{-3}	81.4620	81.9848	0.5228	104.56	18.2235

Table S7

The optimized values of gold thin film, silicon nanosheet thickness and the number of $MoSe_2$ layers with corresponding change in resonance angle and FWHM in SPR curve for 633nm excitation wavelength.

Au thickness (nm)	Si thickness (nm)	Number of $MoSe_2$ layers (L)	Minimum Reflectivity	θ_{SPR} w/o biomolecules (Deg)	θ_{SPR} with biomolecules (Deg)	$\Delta\theta_{SPR}$ (Δn_{bio}=0.005) (Deg)	Sensitivity (Deg/RIU)	FWHM (Deg)
30	0	7	1.6289×10^{-4}	66.2496	66.6354	0.3858	77.16	23.3425
35	0	4	5.0453×10^{-4}	60.6540	60.9799	0.3259	65.18	13.5831
40	0	2	1.7684×10^{-3}	58.0731	58.3531	0.2800	56.00	6.3803
50	0	0	1.4822×10^{-5}	56.1871	56.4280	0.2409	48.18	2.1004
30	5	5	5.5646×10^{-3}	77.1007	77.5029	0.4022	80.44	23.4522
35	5	3	1.9344×10^{-5}	71.7805	72.3006	0.5201	104.02	20.7472
40	5	2	2.2213×10^{-3}	68.9529	69.4724	0.5195	103.90	16.6804
50	5	0	5.0702×10^{-4}	63.7243	64.1544	0.4301	86.02	5.9887
30	7	3	2.5112×10^{-3}	79.2825	79.7482	0.4657	93.14	21.5461
35	7	2	3.8936×10^{-4}	77.2114	77.8079	0.5965	119.30	20.0590
40	7	1	2.0438×10^{-3}	74.0203	74.6787	0.6584	131.68	17.0915
50	7	0	5.5667×10^{-3}	70.7232	71.3733	0.6501	130.02	10.1617

Table S8

The optimized values of gold thin film, silicon nanosheet thickness and the number of $MoSe_2$ layers with corresponding change in resonance angle and FWHM in SPR curve for 660nm excitation wavelength.

Au thickness (nm)	Si thickness (nm)	Number of $MoSe_2$ layers (L)	Minimum Reflectivity	θ_{SPR} w/o biomolecules (Deg)	θ_{SPR} with biomolecules (Deg)	$\Delta\theta_{SPR}$ (Δn_{bio}=0.005) (Deg)	Sensitivity (Deg/RIU)	FWHM (Deg)
30	0	8	2.0024×10^{-5}	65.7655	66.1360	0.3705	74.10	23.0104
35	0	5	4.1023×10^{-9}	60.6852	61.0023	0.3171	63.42	14.1632
40	0	3	7.0439×10^{-4}	58.2607	58.5362	0.2755	55.10	7.1902
50	0	0	2.9483×10^{-4}	55.7750	55.9976	0.2226	44.52	1.9505
30	5	6	4.9718×10^{-4}	75.5255	75.9472	0.4217	84.34	23.6809
35	5	4	1.6159×10^{-4}	70.5878	71.0686	0.4808	96.16	20.8662
40	5	2	5.4002×10^{-4}	65.6024	66.0354	0.4330	86.60	13.8320
50	5	0	4.7825×10^{-4}	61.7097	62.0757	0.3660	73.20	4.9425
30	7	4	7.5071×10^{-3}	77.1916	77.6631	0.4715	94.30	22.2971
35	7	3	9.8331×10^{-5}	75.1079	75.6525	0.5446	108.92	20.7023
40	7	2	1.4928×10^{-3}	72.3527	72.9287	0.5760	115.20	17.9080
50	7	0	1.4158×10^{-3}	66.5248	67.0197	0.4949	98.98	7.8452

Table S9

The optimized values of gold thin film, silicon nanosheet thickness and the number of $MoSe_2$ layers with corresponding change in resonance angle and FWHM in SPR curve for 785nm excitation wavelength.

Au thickness (nm)	Si thickness (nm)	Number of $MoSe_2$ layers (L)	Minimum Reflectivity	θ_{SPR} w/o biomolecules (Deg)	θ_{SPR} with biomolecules (Deg)	$\Delta\theta_{SPR}$ (Δn_{bio}=0.005) (Deg)	Sensitivity (Deg/RIU)	FWHM (Deg)
30	0	12	6.9696×10^{-5}	63.8125	64.1238	0.3113	62.26	22.2247
35	0	7	1.2317×10^{-5}	58.4074	58.6481	0.2407	48.14	11.1258
40	0	4	8.6083×10^{-4}	56.3328	56.5346	0.2018	40.36	4.9499
50	0	0	1.0244×10^{-2}	54.4952	54.6566	0.1614	32.28	1.4493
30	5	11	3.2961×10^{-5}	71.7805	72.1464	0.3659	73.18	24.0905
35	5	6	4.0347×10^{-4}	63.2847	63.6101	0.3254	65.08	16.9494
40	5	3	6.4830×10^{-5}	59.7064	59.9776	0.2712	54.24	8.4272
50	5	0	8.3559×10^{-3}	57.1765	57.3998	0.2233	44.66	2.6852
30	7	10	6.6560×10^{-6}	74.5006	74.8712	0.3706	74.12	23.7053
35	7	6	1.3075×10^{-4}	66.8284	67.2055	0.3771	75.42	19.4897
40	7	3	1.2280×10^{-5}	62.1570	62.4760	0.3190	63.80	11.2734
50	7	0	7.3315×10^{-3}	58.8242	59.0838	0.2596	51.92	3.5672

Table S10

The optimized values of gold thin film, silicon nanosheet thickness and the number of $MoSe_2$ layers with corresponding change in resonance angle and FWHM in SPR curve for 1024nm excitation wavelength.

Au thickness (nm)	Si thickness (nm)	Number of $MoSe_2$ layers (L)	Minimum Reflectivity	θ_{SPR} w/o biomolecules (Deg)	θ_{SPR} with biomolecules (Deg)	$\Delta\theta_{SPR}$ (Δn_{bio}=0.005) (Deg)	Sensitivity (Deg/RIU)	FWHM (Deg)
30	0	37	2.9735×10^{-7}	77.3629	77.6634	0.3005	60.10	22.2105
35	0	15	2.6734×10^{-6}	57.3488	57.5279	0.1791	35.82	8.0993
40	0	3	2.8889×10^{-5}	53.9991	54.1124	0.1133	22.66	1.9736
30	5	32	1.3502×10^{-5}	79.3635	79.6543	0.2908	58.16	21.1464
35	5	15	3.4001×10^{-6}	60.5473	60.7810	0.2337	46.74	13.5613
40	5	3	3.8306×10^{-5}	55.3581	55.5002	0.1421	28.42	3.0126
30	7	30	6.6588×10^{-6}	80.1581	80.4423	0.2842	56.84	20.6365
35	7	15	5.9800×10^{-6}	62.3205	62.5821	0.2616	52.32	15.8192
40	7	3	6.5065×10^{-5}	56.0704	56.2266	0.1562	31.24	3.6162

Table S11
The optimized values of gold thin film, silicon nanosheet thickness and the number of MoS_2 layers with corresponding change in resonance angle and FWHM in SPR curve for 600nm excitation wavelength.

Au thickness (nm)	Si thickness (nm)	Number of MoS_2 layers (L)	Minimum Reflectivity	θ_{SPR} w/o biomolecules (Deg)	θ_{SPR} with biomolecules (Deg)	$\Delta\theta_{SPR}$ (Δn_{bio}=0.005) (Deg)	Sensitivity (Deg/RIU)	FWHM (Deg)
30	0	6	3.8765×10^{-4}	64.3065	64.6843	0.3778	75.56	21.9370
35	0	4	1.9345×10^{-3}	61.1528	61.5006	0.3478	69.56	14.5927
40	0	2	1.5126×10^{-5}	58.6783	58.9833	0.3050	61.00	7.1320
50	0	0	6.0974×10^{-5}	56.7966	57.0636	0.2670	53.40	2.3134
30	5	4	6.1501×10^{-3}	76.8775	77.3078	0.4303	86.06	23.3656
35	5	2	2.6005×10^{-3}	72.4389	73.0104	0.5715	114.30	20.0467
40	5	1	3.1793×10^{-3}	69.9714	70.5499	0.5785	115.70	15.4579
50	5	0	2.6443×10^{-3}	67.5897	68.1553	0.5656	113.12	8.0491
30	7	2	9.6041×10^{-5}	81.2746	81.7083	0.4337	86.74	20.2786
35	7	1	1.7734×10^{-4}	80.5325	81.1722	0.6397	127.94	18.4122

Table S12
The optimized values of gold thin film, silicon nanosheet thickness and the number of MoS_2 layers with corresponding change in resonance angle and FWHM in SPR curve for 633nm excitation wavelength.

Au thickness (nm)	Si thickness (nm)	Number of MoS_2 layers (L)	Minimum Reflectivity	θ_{SPR} w/o biomolecules (Deg)	θ_{SPR} with biomolecules (Deg)	$\Delta\theta_{SPR}$ (Δn_{bio}=0.005) (Deg)	Sensitivity (Deg/RIU)	FWHM (Deg)
30	0	6	1.1588×10^{-5}	65.0423	65.4132	0.3709	74.18	22.9767
35	0	4	9.8118×10^{-4}	61.0689	61.4022	0.3333	66.66	15.2011
40	0	2	3.3624×10^{-6}	58.2075	58.4904	0.2829	56.58	6.8984
50	0	0	1.4822×10^{-5}	56.1871	56.4280	0.2409	48.18	2.1004
30	5	4	3.7343×10^{-6}	75.1781	75.6259	0.4478	89.56	23.6669
35	5	3	3.8228×10^{-3}	72.6068	73.1200	0.5132	102.64	21.5806
40	5	2	9.6549×10^{-3}	69.5062	70.0324	0.5262	105.24	17.6990
50	5	0	5.0702×10^{-4}	63.7243	64.1544	0.4301	86.02	5.9887
30	7	3	1.5195×10^{-3}	79.7645	80.1558	0.3913	78.26	21.8300
35	7	2	2.5629×10^{-3}	77.7725	78.3248	0.5523	110.46	20.4299
40	7	1	1.1513×10^{-5}	74.4025	75.0610	0.6586	131.72	17.5728
50	7	0	5.5667×10^{-3}	70.7232	71.3733	0.6501	130.02	10.1617

Table S13

The optimized values of gold thin film, silicon nanosheet thickness and the number of MoS_2 layers with corresponding change in resonance angle and FWHM in SPR curve for 660nm excitation wavelength.

Au thickness (nm)	Si thickness (nm)	Number of MoS_2 layers (L)	Minimum Reflectivity	θ_{SPR} w/o biomolecules (Deg)	θ_{SPR} with biomolecules (Deg)	$\Delta\theta_{SPR}$ (Δn_{bio}=0.005) (Deg)	Sensitivity (Deg/RIU)	FWHM (Deg)
30	0	6	1.8860×10^{-4}	63.0347	63.3709	0.3362	67.24	21.6314
35	0	4	4.3030×10^{-4}	59.7976	60.0976	0.3000	60.00	13.5477
40	0	2	2.7099×10^{-5}	57.4727	57.7309	0.2582	51.64	6.1343
50	0	0	2.9483×10^{-4}	55.7750	55.9976	0.2226	44.52	1.9505
30	5	5	1.5371×10^{-3}	73.8012	74.2142	0.4130	82.60	24.3312
35	5	3	3.3096×10^{-6}	68.5553	69.0066	0.4513	90.26	20.2592
40	5	2	3.5910×10^{-3}	66.0163	66.4559	0.4396	87.92	15.3721
50	5	0	4.7825×10^{-4}	61.7097	62.0757	0.3660	73.20	4.9425
30	7	4	2.4965×10^{-3}	77.6969	78.0907	0.3938	78.76	23.1151
35	7	2	6.6891×10^{-3}	72.3108	72.8323	0.5215	104.30	20.1227
40	7	1	6.5220×10^{-3}	69.3471	69.8655	0.5184	103.68	15.4924
50	7	0	1.4158×10^{-3}	66.5248	67.0197	0.4949	98.98	7.8452

Table S14

The optimized values of gold thin film, silicon nanosheet thickness and the number of MoS_2 layers with corresponding change in resonance angle and FWHM in SPR curve for 785nm excitation wavelength.

Au thickness (nm)	Si thickness (nm)	Number of MoS_2 layers (L)	Minimum Reflectivity	θ_{SPR} w/o biomolecules (Deg)	θ_{SPR} with biomolecules (Deg)	$\Delta\theta_{SPR}$ (Δn_{bio}=0.005) (Deg)	Sensitivity (Deg/RIU)	FWHM (Deg)
30	0	21	4.1054×10^{-3}	85.4520	85.6781	0.2261	45.22	15.6719
35	0	19	8.0650×10^{-3}	81.8865	82.3516	0.4651	93.02	18.1127
40	0	18	1.6636×10^{-3}	79.4828	80.0256	0.5428	108.56	18.2150
50	0	0	1.0244×10^{-2}	54.4952	54.6566	0.1614	32.28	1.4493
30	5	15	1.9347×10^{-2}	85.4900	85.7774	0.2874	57.48	15.1351
35	5	14	1.7224×10^{-3}	84.3663	84.6635	0.2972	59.44	15.8351
40	5	12	1.5890×10^{-3}	78.7526	79.3507	0.5981	119.62	17.5634
50	5	0	8.3559×10^{-3}	57.1765	57.3998	0.2233	44.66	2.6852
30	7	13	6.0457×10^{-3}	86.1192	86.3435	0.2243	44.86	14.0449
35	7	12	1.1254×10^{-2}	85.0190	85.2241	0.2051	41.02	14.9529
40	7	10	1.4479×10^{-3}	79.7251	80.3357	0.6106	122.12	17.0554
50	7	0	7.3315×10^{-3}	58.8242	59.0838	0.2596	51.92	3.5672

Table S15

The optimized values of gold thin film, silicon nanosheet thickness and the number of MoS_2 layers with corresponding change in resonance angle and FWHM in SPR curve for 1024nm excitation wavelength.

Au thickness (nm)	Si thickness (nm)	Number of MoS_2 layers (L)	Minimum Reflectivity	θ_{SPR} w/o biomolecules (Deg)	θ_{SPR} with biomolecules (Deg)	$\Delta\theta_{SPR}$ (Δn_{bio}=0.005) (Deg)	Sensitivity (Deg/RIU)	FWHM (Deg)
30	0	37	3.7590×10^{-4}	86.6058	86.7180	0.1122	22.44	13.1950
35	0	34	3.9641×10^{-3}	83.4687	83.7908	0.3221	64.42	16.6822
40	0	24	6.6910×10^{-7}	64.8861	65.2007	0.3146	62.92	15.7915
30	5	31	3.5648×10^{-4}	86.7583	86.8695	0.1112	22.24	12.6806
35	5	29	2.9629×10^{-3}	85.0857	85.2971	0.2114	42.28	14.8210
40	5	26	1.6573×10^{-6}	79.6388	80.0887	0.4499	89.98	17.5445
30	7	29	9.2532×10^{-3}	86.9999	87.0640	0.0641	12.82	12.0259
35	7	26	3.2804×10^{-3}	84.1379	84.4549	0.3170	63.40	15.6223
40	7	24	2.6466×10^{-5}	80.4023	80.8507	0.4484	89.68	17.0995

Table S16

The optimized values of gold thin film, silicon nanosheet thickness and the number of WSe_2 layers with corresponding change in resonance angle and FWHM in SPR curve for 600nm excitation wavelength.

Au thickness (nm)	Si thickness (nm)	Number of WSe_2 layers (L)	Minimum Reflectivity	θ_{SPR} w/o biomolecules (Deg)	θ_{SPR} with biomolecules (Deg)	$\Delta\theta_{SPR}$ (Δn_{bio}=0.005) (Deg)	Sensitivity (Deg/RIU)	FWHM (Deg)
30	0	6	1.3325×10^{-3}	65.9641	66.3667	0.4026	80.52	22.2635
35	0	4	9.8589×10^{-5}	61.9820	62.3467	0.3647	72.94	14.8483
40	0	2	1.4661×10^{-3}	58.9733	59.2852	0.3119	62.38	7.1293
50	0	0	6.0974×10^{-5}	56.7966	57.0636	0.2670	53.40	2.3134
30	5	4	8.8668×10^{-3}	79.1252	79.5096	0.3844	76.88	22.3640
35	5	2	6.5971×10^{-3}	73.6359	74.2378	0.6019	120.38	19.8777
40	5	1	7.0381×10^{-3}	70.5039	71.0993	0.5954	119.08	15.4087
50	5	0	2.6443×10^{-3}	67.5897	68.1553	0.5656	113.12	8.0491
30	7	2	5.6634×10^{-4}	82.6512	83.0077	0.3565	71.30	19.1634
35	7	1	1.1040×10^{-5}	81.4876	82.0996	0.6120	122.40	17.8356

Table S17
The optimized values of gold thin film, silicon nanosheet thickness and the number of WSe_2 layers with corresponding change in resonance angle and FWHM in SPR curve for 633nm excitation wavelength.

Au thickness (nm)	Si thickness (nm)	Number of WSe_2 layers (L)	Minimum Reflectivity	θ_{SPR} w/o biomolecules (Deg)	θ_{SPR} with biomolecules (Deg)	$\Delta\theta_{SPR}$ (Δn_{bio}=0.005) (Deg)	Sensitivity (Deg/RIU)	FWHM (Deg)
30	0	11	2.2768×10^{-3}	78.5367	78.9284	0.3917	78.34	22.8451
35	0	8	5.8035×10^{-4}	69.4428	69.9257	0.4829	96.58	20.4245
40	0	5	6.9205×10^{-5}	62.2455	62.6176	0.3721	74.42	11.1533
50	0	0	1.4822×10^{-5}	56.1871	56.4280	0.2409	48.18	2.1004
30	5	6	2.2033×10^{-3}	82.4372	82.7518	0.3146	62.92	19.6486
35	5	4	9.6397×10^{-3}	76.3932	77.0125	0.6193	123.86	19.7620
40	5	3	3.3950×10^{-4}	72.9343	73.5571	0.6228	124.56	17.3262
50	5	0	5.0702×10^{-4}	63.7243	64.1544	0.4301	86.02	5.9887
30	7	4	3.3021×10^{-3}	84.1284	84.3689	0.2405	48.10	17.5654
35	7	3	1.1646×10^{-2}	82.5553	82.9505	0.3952	79.04	17.5903
40	7	2	5.3807×10^{-3}	79.5347	80.2417	0.7070	141.40	17.2340
50	7	0	5.5667×10^{-3}	70.7232	71.3733	0.6501	130.02	10.7091

Table S18
The optimized values of gold thin film, silicon nanosheet thickness and the number of WSe_2 layers with corresponding change in resonance angle and FWHM in SPR curve for 660nm excitation wavelength.

Au thickness (nm)	Si thickness (nm)	Number of WSe_2 layers (L)	Minimum Reflectivity	θ_{SPR} w/o biomolecules (Deg)	θ_{SPR} with biomolecules (Deg)	$\Delta\theta_{SPR}$ (Δn_{bio}=0.005) (Deg)	Sensitivity (Deg/RIU)	FWHM (Deg)
30	0	14	9.9083×10^{-4}	82.0877	82.4070	0.3193	63.86	20.0880
35	0	12	4.3537×10^{-5}	77.0606	77.6036	0.5430	108.60	20.6870
40	0	9	2.4735×10^{-5}	67.8256	68.3062	0.4806	96.12	16.1546
50	0	0	2.9483×10^{-4}	55.7750	55.9976	0.2226	44.52	1.9505
30	5	8	2.8380×10^{-3}	83.5346	83.8918	0.3572	71.44	18.0605
35	5	7	4.3860×10^{-3}	81.8189	82.2685	0.4496	89.92	18.2067
40	5	5	1.0418×10^{-3}	74.7498	75.3946	0.6448	128.96	17.5697
50	5	0	4.7825×10^{-4}	61.7097	62.0757	0.3660	73.20	4.9425
30	7	6	6.2526×10^{-4}	84.9979	85.2229	0.2250	45.00	16.1847
35	7	5	1.8639×10^{-2}	83.5895	83.8794	0.2899	57.98	16.6250
40	7	3	6.8553×10^{-3}	76.5740	77.2831	0.7091	141.82	16.9720
50	7	0	1.4158×10^{-3}	66.5248	67.0197	0.4949	98.98	7.8452

Table S19
The optimized values of gold thin film, silicon nanosheet thickness and the number of WSe_2 layers with corresponding change in resonance angle and FWHM in SPR curve for 785nm excitation wavelength.

Au thickness (nm)	Si thickness (nm)	Number of WSe_2 layers (L)	Minimum Reflectivity	θ_{SPR} w/o biomolecules (Deg)	θ_{SPR} with biomolecules (Deg)	$\Delta\theta_{SPR}$ (Δn_{bio}=0.005) (Deg)	Sensitivity (Deg/RIU)	FWHM (Deg)
30	0	22	8.4871×10^{-3}	87.4001	87.4328	0.0327	6.54	11.0742
35	0	20	6.7847×10^{-3}	85.0150	85.3998	0.3848	76.96	14.6494
40	0	19	3.7420×10^{-4}	82.9005	83.4261	0.5256	105.12	15.6095
50	0	0	1.0244×10^{-2}	54.4952	54.6566	0.1614	32.28	1.4493
30	5	16	3.0767×10^{-4}	87.4542	87.5420	0.0878	17.56	10.8336
35	5	14	2.5920×10^{-2}	84.5495	85.0380	0.4885	97.70	14.7270
40	5	13	3.0089×10^{-3}	82.1762	82.8034	0.6272	125.44	15.5520
50	5	0	8.3559×10^{-3}	57.1765	57.3998	0.2233	44.66	2.6852
30	7	14	2.9648×10^{-2}	87.6125	87.5761	0.0364	7.28	10.2158
35	7	12	6.9144×10^{-3}	85.4400	85.8225	0.3825	76.50	13.7719
40	7	11	1.5685×10^{-7}	83.2732	83.8288	0.5556	111.12	14.8756
50	7	0	7.3315×10^{-3}	58.8242	59.0838	0.2596	51.92	3.5672

Table S20
The optimized values of gold thin film, silicon nanosheet thickness and the number of WSe_2 layers with corresponding change in resonance angle and FWHM in SPR curve for 1024nm excitation wavelength.

Au thickness (nm)	Si thickness (nm)	Number of WSe_2 layers (L)	Minimum Reflectivity	θ_{SPR} w/o biomolecules (Deg)	θ_{SPR} with biomolecules (Deg)	$\Delta\theta_{SPR}$ (Δn_{bio}=0.005) (Deg)	Sensitivity (Deg/RIU)	FWHM (Deg)
30	0	30	1.9402×10^{-3}	83.6259	83.8350	0.2091	41.82	18.6024
35	0	27	7.0563×10^{-5}	78.6477	78.9901	0.3424	68.48	20.8099
40	0	4	3.6825×10^{-5}	54.3710	54.4926	0.1216	24.32	2.3459
30	5	25	1.8613×10^{-3}	84.0992	84.3069	0.2077	41.54	17.6802
35	5	23	6.5693×10^{-4}	81.1789	81.4915	0.3126	62.52	19.1731
40	5	4	4.6665×10^{-5}	55.9801	56.1347	0.1546	30.92	3.7128
30	7	23	2.6872×10^{-3}	84.2168	84.4294	0.2126	42.52	17.3711
35	7	21	1.2616×10^{-4}	81.2364	81.5596	0.3232	64.64	18.9030
40	7	5	2.2124×10^{-5}	57.3452	57.5259	0.1807	36.14	5.2062

2.2. Phase interrogation

In order to further demonstrate the validity of our N-layer 2D models, we also studied the SPR phase signal changes with different refractive-index changes at the sensing surfaces. The thickness of the metallic silicon-MX_2 thin film are optimized under three excitation wavelengths, namely 633 nm, 660 nm, 1540 nm for the phase interrogation scheme. The phase sensitivity can reach to one order of magnitude higher than that of the conventional configuration, as shown in Fig. S8-S10 and Table S21-26.

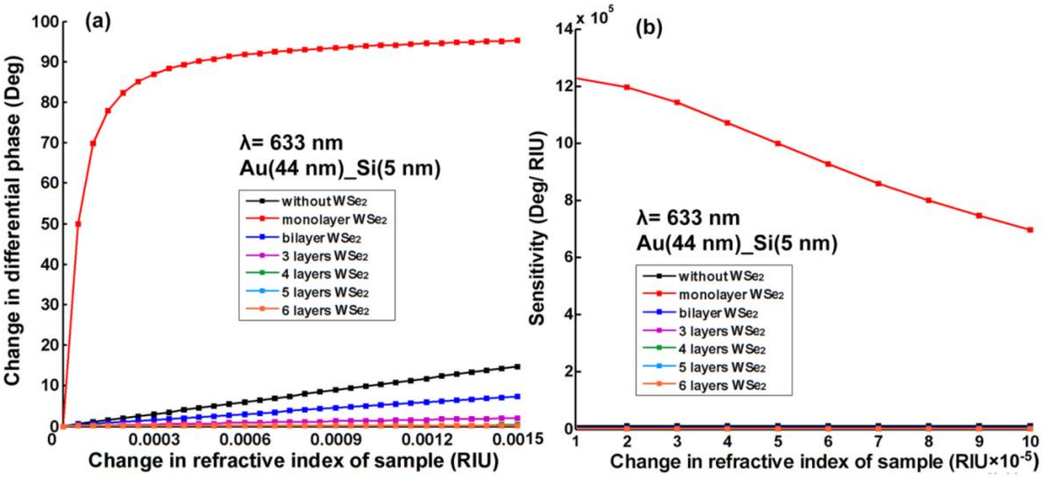

Figure S8. At 633 nm excitation wavelength, in silicon-WSe_2 enhanced model, the optimized thickness are 44 nm gold and 5 nm silicon. (a) The change in differential phase as a function of the refractive index change of the biomolecular sample. (b) The phase sensitvity as a function of the refractive index change of the biomolecular sample that ranges from 1×10^{-5} RIU to 1×10^{-4} RIU.

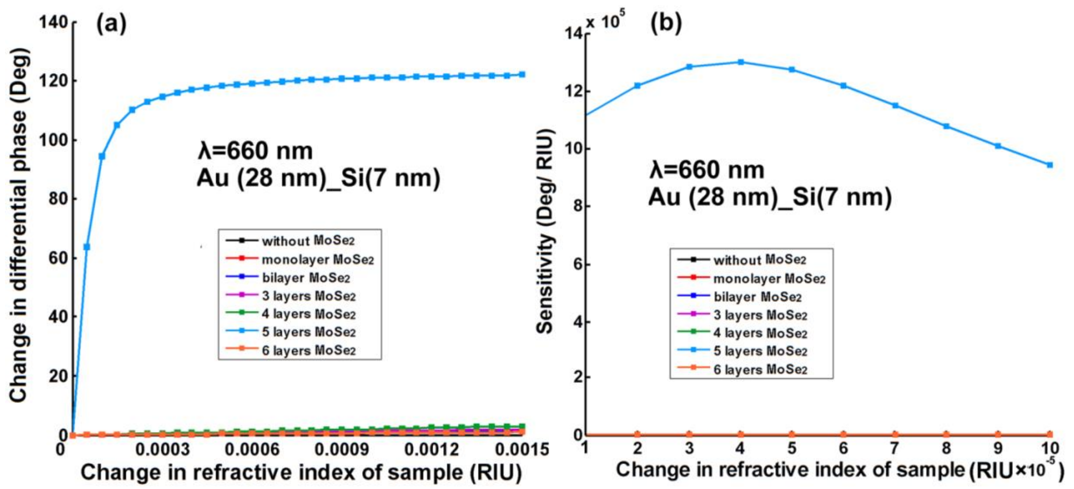

Figure S9. At 660 nm excitation wavelength, in silicon-$MoSe_2$ enhanced model, the optimized thicknesses are 28 nm gold and 7 nm silicon. (a) The change in differential phase as a function of the refractive index change of the biomolecular sample. (b) The phase sensitivity as a function of the refractive index change of the biomolecular sample that ranges from 1×10^{-5} RIU to 1×10^{-4} RIU.

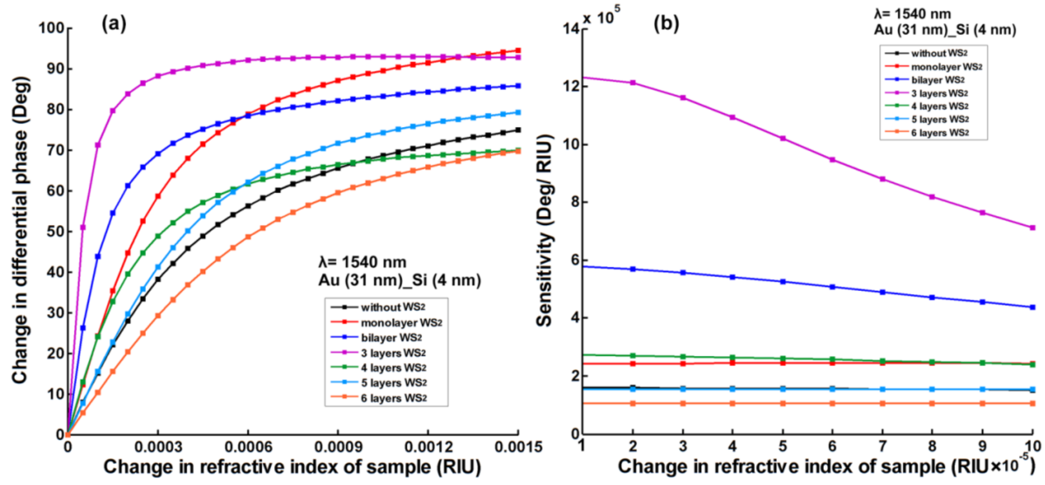

Figure S10. At 1540 nm excitation wavelength, in silicon-WS_2 enhanced model, the optimized thicknesses are 31 nm gold and 4 nm silicon. (a) The change in differential phase as a function of the refractive index change of the biomolecular sample. (b) The phase sensitivity as a function of the refractive index change of the biomolecular sample that ranges from 1×10^{-5} RIU to 1×10^{-4} RIU.

Table S21

The change in differential phase of various refractive index change of the biomolecular sample with optimized thickness of 44 nm gold and 5 nm silicon at 633 nm excitation wavelength in silicon-WSe_2 model.

Number of WSe_2 layers (L)	Change in differential phase (Deg) $\Delta n=1\times10^{-5}$	Change in differential phase (Deg) $\Delta n=3\times10^{-5}$	Change in differential phase (Deg) $\Delta n=5\times10^{-5}$	Change in differential phase (Deg) $\Delta n=7\times10^{-5}$	Change in differential phase (Deg) $\Delta n=9\times10^{-5}$
0	0.0982	0.2949	0.4916	0.6883	0.8851
1	12.2576	34.2615	49.9405	60.2348	67.1041
2	0.0502	0.1507	0.2511	0.3514	0.4517
3	0.0135	0.0404	0.0674	0.0943	0.1212
4	0.0017	0.0052	0.0087	0.0122	0.0157
5	0.0007	0.0023	0.0038	0.0054	0.0070
6	0.0008	0.0026	0.0044	0.0061	0.0079

Table S22

The phase sensitivity of various refractive index change of the biomolecular sample with optimized thickness of 44 nm gold and 5 nm silicon at 633 nm excitation wavelength in silicon-WSe_2 model.

Number of WSe_2 layers (L)	Phase sensitivity (Deg/RIU) $\Delta n=1\times10^{-5}$	Phase sensitivity (Deg/RIU) $\Delta n=3\times10^{-5}$	Phase sensitivity (Deg/RIU) $\Delta n=5\times10^{-5}$	Phase sensitivity (Deg/RIU) $\Delta n=7\times10^{-5}$	Phase sensitivity (Deg/RIU) $\Delta n=9\times10^{-5}$
0	9.8295×10^3	9.8311×10^3	9.8325×10^3	9.8340×10^3	9.8353×10^3
1	1.2258×10^6	1.1421×10^6	9.9881×10^5	8.6050×10^5	7.4560×10^5
2	5.0264×10^3	5.0246×10^3	5.0228×10^3	5.0209×10^3	5.0191×10^3
3	1.3500×10^3	1.3494×10^3	1.3488×10^3	1.3482×10^3	1.3476×10^3
4	1.7554×10^2	1.7537×10^2	1.7521×10^2	1.7505×10^2	1.7489×10^2
5	7.7862×10^1	7.7883×10^1	7.7904×10^1	7.7925×10^1	7.7946×10^1
6	8.8553×10^1	8.8553×10^1	8.8553×10^1	8.8553×10^1	8.8553×10^1

Table S23

The change in differential phase of various refractive index change of the biomolecular sample with optimized thickness of 28 nm gold, 7 nm silicon at 660 nm excitation wavelength in silicon-$MoSe_2$ model.

Number of $MoSe_2$ layers (L)	Change in differential phase (Deg) $\Delta n=1\times10^{-5}$	Change in differential phase (Deg) $\Delta n=3\times10^{-5}$	Change in differential phase (Deg) $\Delta n=5\times10^{-5}$	Change in differential phase (Deg) $\Delta n=7\times10^{-5}$	Change in differential phase (Deg) $\Delta n=9\times10^{-5}$
0	0.0105	0.0317	0.0528	0.0739	0.0951
1	0.0107	0.0321	0.0535	0.0749	0.0964
2	0.0113	0.0339	0.0566	0.0793	0.1019
3	0.0132	0.0397	0.0662	0.0927	0.1192
4	0.0199	0.0598	0.0997	0.1397	0.1796
5	11.1660	38.5214	63.7063	80.4979	90.8135
6	0.0072	0.0216	0.0360	0.0504	0.0648

Table S24

The phase sensitivity of various refractive index change of the biomolecular sample with optimized thickness of 28 nm gold and 7 nm silicon at 660 nm excitation wavelength in silicon-$MoSe_2$ model.

Number of $MoSe_2$ layers (L)	Phase sensitivity (Deg/RIU) $\Delta n=1\times10^{-5}$	Phase sensitivity (Deg/RIU) $\Delta n=3\times10^{-5}$	Phase sensitivity (Deg/RIU) $\Delta n=5\times10^{-5}$	Phase sensitivity (Deg/RIU) $\Delta n=7\times10^{-5}$	Phase sensitivity (Deg/RIU) $\Delta n=9\times10^{-5}$
0	1.0567×10^{3}	1.0567×10^{3}	1.0568×10^{3}	1.0568×10^{3}	1.0569×10^{3}
1	1.0710×10^{3}	1.0711×10^{3}	1.0711×10^{3}	1.0712×10^{3}	1.0712×10^{3}
2	1.1328×10^{3}	1.1329×10^{3}	1.1330×10^{3}	1.1330×10^{3}	1.1331×10^{3}
3	1.3247×10^{3}	1.3248×10^{3}	1.3249×10^{3}	1.3250×10^{3}	1.3252×10^{3}
4	1.9947×10^{3}	1.9952×10^{3}	1.9957×10^{3}	1.9961×10^{3}	1.9966×10^{3}
5	1.1166×10^{6}	1.2840×10^{6}	1.2741×10^{6}	1.1500×10^{6}	1.0090×10^{6}
6	7.2197×10^{2}	7.2154×10^{2}	7.2111×10^{2}	7.2068×10^{2}	7.2025×10^{2}

Table S25
The change in differential phase of various refractive index change of the biomolecular sample with optimized thickness of 31 nm gold and 4 nm silicon at 1540 nm excitation wavelength in silicon-WS_2 model.

Number of WS_2 layers (L)	Change in differential phase (Deg) $\Delta n=1\times10^{-5}$	Change in differential phase (Deg) $\Delta n=3\times10^{-5}$	Change in differential phase (Deg) $\Delta n=5\times10^{-5}$	Change in differential phase (Deg) $\Delta n=7\times10^{-5}$	Change in differential phase (Deg) $\Delta n=9\times10^{-5}$
0	1.5913	4.7404	7.8307	10.8474	13.7776
1	2.4114	7.3091	12.2496	17.1620	21.9780
2	5.7761	16.6771	26.2493	34.3003	40.9254
3	12.3201	34.8428	51.0375	61.6276	68.6356
4	2.7394	8.0375	13.0367	17.6907	21.9772
5	1.5417	4.6442	7.7562	10.8589	13.9335
6	1.0446	3.1367	5.2279	7.3121	9.3837

Table S26
The phase sensitivity of various refractive index change of the biomolecular sample with optimized thickness of 31 nm gold and 4 nm silicon at 1540 nm excitation wavelength in silicon-WS_2 model.

Number of WS_2 layers (L)	Phase sensitivity (Deg/RIU) $\Delta n=1\times10^{-5}$	Phase sensitivity (Deg/RIU) $\Delta n=3\times10^{-5}$	Phase sensitivity (Deg/RIU) $\Delta n=5\times10^{-5}$	Phase sensitivity (Deg/RIU) $\Delta n=7\times10^{-5}$	Phase sensitivity (Deg/RIU) $\Delta n=9\times10^{-5}$
0	1.5914×10^5	1.5802×10^5	1.5662×10^5	1.5496×10^5	1.5308×10^5
1	2.4115×10^5	2.4364×10^5	2.4499×10^5	2.4517×10^5	2.4420×10^5
2	5.7762×10^5	5.5590×10^5	5.2499×10^5	4.9001×10^5	4.5473×10^5
3	1.2320×10^6	1.1614×10^6	1.0208×10^6	8.8040×10^5	7.6262×10^5
4	2.7394×10^5	2.6792×10^5	2.6074×10^5	2.5272×10^5	2.4419×10^5
5	1.5418×10^5	1.5481×10^5	1.5513×10^5	1.5513×10^5	1.5482×10^5
6	1.0446×10^5	1.0456×10^5	1.0456×10^5	1.0446×10^5	1.0426×10^5

Table S27
Comparison of the 2D MX_2-enhanced SPR sensing performances with those of current state-of-art SPR biosensors.

**Detection Scheme**	**Measured Signal**	**Excitation Wavelength (nm)**	**Enhanced Mechanism**	**System configuration**	**Sensitivity Definition**	**Sensitivity** (Deg/RIU)	**Ref.**
Angular interrogation	θ_{SPR}	633	SF14 prism with gold substrate	K. C.	$\frac{d\theta_{SPR}}{dn}$	~70	[*Sensor Actuat B-Chem 54, 16, (1999)*]
Angular interrogation	θ_{SPR}	532	SF11 prism with silver substrate	K. C.	$\frac{d\theta_{SPR}}{dn}$	~90	[*J Opt a-Pure Appl Op 8, 959, (2006)*]
Angular interrogation	θ_{SPR}	600	Metallic Si-WS_2 thin film	K. C.	$\frac{d\theta_{SPR}}{dn}$	~155	supplementary Table S1
Phase interrogation	Phase	1500	Silicon based prism	K. C.	$\frac{d(\varphi_{TM}-\varphi_{TE})}{dn}$	~3×10^3	[*Opt. Express 17, 20847, (2009)*]
Phase interrogation	Phase	633	SPR-interferometer	K. C.	$\frac{d(\varphi_{TM}-\varphi_{TE})}{dn}$	1.5×10^5	[*Opt. Commun. 150, 5, (1998)*]
Phase interrogation	Phase	633	Mach-Zehnder interferometer	interferometer	$\frac{d(\varphi_{TM}-\varphi_{TE})}{dn}$	1.8×10^5	[*Opt. Express 17, 21191, (2009)*]
Phase interrogation	Phase	633	Polarimetry	PEM- based polarimeter	$\frac{d(\varphi_{TM}-\varphi_{TE})}{dn}$	1.2×10^5	[*Sensor Actuat a-Phys 151, 23, (2009)*]
Phase interrogation	Phase	633	Metallic Si-WSe_2 thin film	K. C. and interferometer	$\frac{d(\varphi_{TM}-\varphi_{TE})}{dn}$	1.2×10^6	supplementary Table S22
Phase interrogation	Phase	660	Metallic Si-$MoSe_2$ thin film	K. C. and interferometer	$\frac{d(\varphi_{TM}-\varphi_{TE})}{dn}$	1.1×10^6	supplementary Table S24
Phase interrogation	Phase	1540	Metallic Si-WS_2 thin film	K. C. and interferometer	$\frac{d(\varphi_{TM}-\varphi_{TE})}{dn}$	1.2×10^6	supplementary Table S26
